# Incidence and influencing factors of surgical site infection in patients with oral cancer: a systematic review and meta-analysis

**DOI:** 10.3389/fonc.2026.1796067

**Published:** 2026-05-08

**Authors:** Xin-Ya Qin, Jie-Bin Yang, Ya-Mei Liu, Li-Li Hou

**Affiliations:** 1 College of Nursing, Chengdu University of Traditional Chinese Medicine, Chengdu, China; 2 Department of Nursing, Affiliated Ninth People’s Hospital, Shanghai Jiao Tong University School of Medicine, Shanghai, China

**Keywords:** incidence, meta-analysis, oral cancer, risk factors, surgical site infection

## Abstract

**Background:**

The incidence and influencing factors of surgical site infection(SSI) in oral cancer were investigated through meta-analysis.

**Methods:**

The search was conducted from inception until April 2024 in databases including PubMed, Embase, Web of Science, and Cochrane Library using a combination of subject terms and keywords. Observational studies (cohort and case-control) investigating the incidence and influencing factors of SSI in patients with oral cancer were included for data collection. Quality assessment was performed, followed by extraction and analysis of valid data using Stata 17.0 software.

**Results:**

The pooled incidence of SSI was 29% (95% CI: 25%–33%) with substantial heterogeneity. Factors associated with SSI included radiotherapy, mandibulectomy, diabetes, ASA classification, operative time, reconstructive surgery, transfusion, neck dissection, and albumin.

**Conclusion:**

These factors were associated with SSI in oral cancer patients and may help identify high-risk individuals.

**Systematic Review Registration:**

https://www.crd.york.ac.uk/PROSPERO/, identifier CRD42024539962.

## 1 Introduction

Oral cancer is defined as the existence of malignant neoplasms in the oral cavity and its adjacent anatomical structures. Tumors primarily occur in anatomic locations such as the lip, tongue, maxillary gum, posterior triangle of molar teeth, floor of mouth, hard palate, and anterior two-thirds of the tongue. Incidence and mortality rates have been consistently increasing over time. The Global Cancer Statistics for 2022 reveal a total of 389,485 newly diagnosed cases of oral cancer and an overall mortality rate of 188,230 deaths (1). The current treatment for oral cancer primarily involves surgical intervention. However, there has been an increase in the duration of surgery and blood loss associated with oral cancer procedures. Additionally, these surgeries are highly invasive and can lead to severe postoperative complications, with the most common being surgical site infections (SSI) (2, 3). Although appropriate antibiotic administration and aggressive surgical debridement can effectively prevent the occurrence of surgical site infections (4), the invasive nature of oral cancer surgery, such as neck dissection for oral cancer patients, can create additional communication pathways between the neck and mouth. This subsequently increases the potential for migration of native oral bacteria to the neck area, thereby leading to an increased risk of surgical site infection (5). SSI is one of the most prevalent healthcare-associated infections (6). Its occurrence not only has a detrimental impact on patients’ physical and mental well-being, as well as hospital resources, but also significantly amplifies the clinical and economic challenges associated with surgery. Globally, SSI is associated with an elevated incidence of morbidity and mortality, leading to various consequences including escalated surgical interventions, diminished quality of life, prolonged antibiotic usage and recovery duration, as well as subsequent impairment in work productivity.

By conducting a comprehensive literature review and employing the research methodology of meta-analysis, this study primarily synthesized the incidence rates and influential factors associated with surgical site infections, thereby offering valuable insights for further exploration of pertinent preventive and intervention measures in subsequent investigations. A recent meta-analysis by Chen et al. also investigated risk factors for SSI in oral cancer surgery. Building on that work, the present study incorporates more recent evidence and a larger sample to provide a more comprehensive and updated synthesis.

## 2 Materials and methods

### 2.1 Literature retrieval strategy

This study followed the PRISMA checklist guidelines. The study was registered on PROSPERO on April 25th, 2024, with the registration number CRD42024539962. The literature search was conducted in PubMed, Cochrane Library, Embase, and Web of Science databases from inception to April 26, 2024. Only studies published in English were included. In addition to database searching, the reference lists of relevant studies were manually screened. Grey literature was not systematically searched. **Supplementary Tables 1**-**4** presents the comprehensive search strategy employed for each database.

### 2.2 Inclusion and exclusion criteria

Inclusion criteria: 1) Participants: patients diagnosed with oral cancer who underwent surgical treatment; 2) Study design: observational studies (cohort or case-control); 3) Outcome: reported surgical site infection (SSI); 4) Data: provided sufficient data to extract odds ratios (ORs) with 95% confidence intervals; 5) Publication: full-text articles published in English. Exclusion criteria: 1) were reviews, case reports, conference abstracts, or non-original studies; 2) did not report SSI as an outcome; 3) did not provide sufficient data for analysis; 4) reported infections unrelated to surgical site infections; 5) involved non-oral cancer populations or non-surgical treatments.

### 2.3 Study selection and data extraction

Two researchers independently screened studies against the inclusion and exclusion criteria, resolving discrepancies through discussion or by consulting a third reviewer. For missing or ambiguous data, corresponding authors were contacted. The extracted information included: first author, year of publication, study type, country, timing of surgery, total sample size, number of infections, male-to-female ratio, age range, SSI definition, and significant factors.

### 2.4 Quality evaluation of included studies

Study quality was assessed using the Newcastle-Ottawa Scale (NOS) for cohort and case-control studies (7).The NOS scale encompasses subject selection, comparability between groups, as well as outcome or exposure. One point is awarded for each met criterion. The total possible score is 9. Studies scoring ≥7, 4-6, and ≤3 were considered high, medium, and low quality, respectively. Two researchers independently performed the assessments, with a third resolving disagreements. All included studies met acceptable quality standards based on NOS assessment.

### 2.5 Statistical analysis

Statistical analyses were performed using Stata version 17.0. A two-sided P value < 0.05 was considered statistically significant. Pooled odds ratios (ORs) with 95% confidence intervals (CIs) were calculated. Heterogeneity was assessed using the I² statistic. A fixed-effects model was applied when I² < 50%; otherwise, a random-effects model was used. Both cohort and case-control studies were included. All included studies reported odds ratios, allowing quantitative synthesis across study designs. Effect estimates were extracted from multivariable logistic regression analyses reported in the original studies. However, the adjustment for confounders varied across studies. Operative time was analyzed according to reporting format. Studies reporting categorical operative time (e.g., ≥6 h vs <6 h) were pooled using meta-analysis. Studies reporting operative time as continuous variables were not pooled due to inconsistent measurement units (e.g., minutes vs hours) and were summarized descriptively. Radiotherapy was extracted as reported, including preoperative, postoperative, or unspecified prior radiotherapy, and was analyzed as a single variable. Sensitivity analyses were performed to assess the stability of the results. Publication bias was evaluated using funnel plots for subgroups with ≥10 studies and Egger’s or Begg’s tests for subgroups with fewer than 10 studies. Study quality was assessed using the Newcastle–Ottawa Scale (NOS). Studies were not excluded based on quality scores, and quality assessment was used to evaluate potential risk of bias.

## 3 Results

### 3.1 Results of the literature search

**Figure 1:**
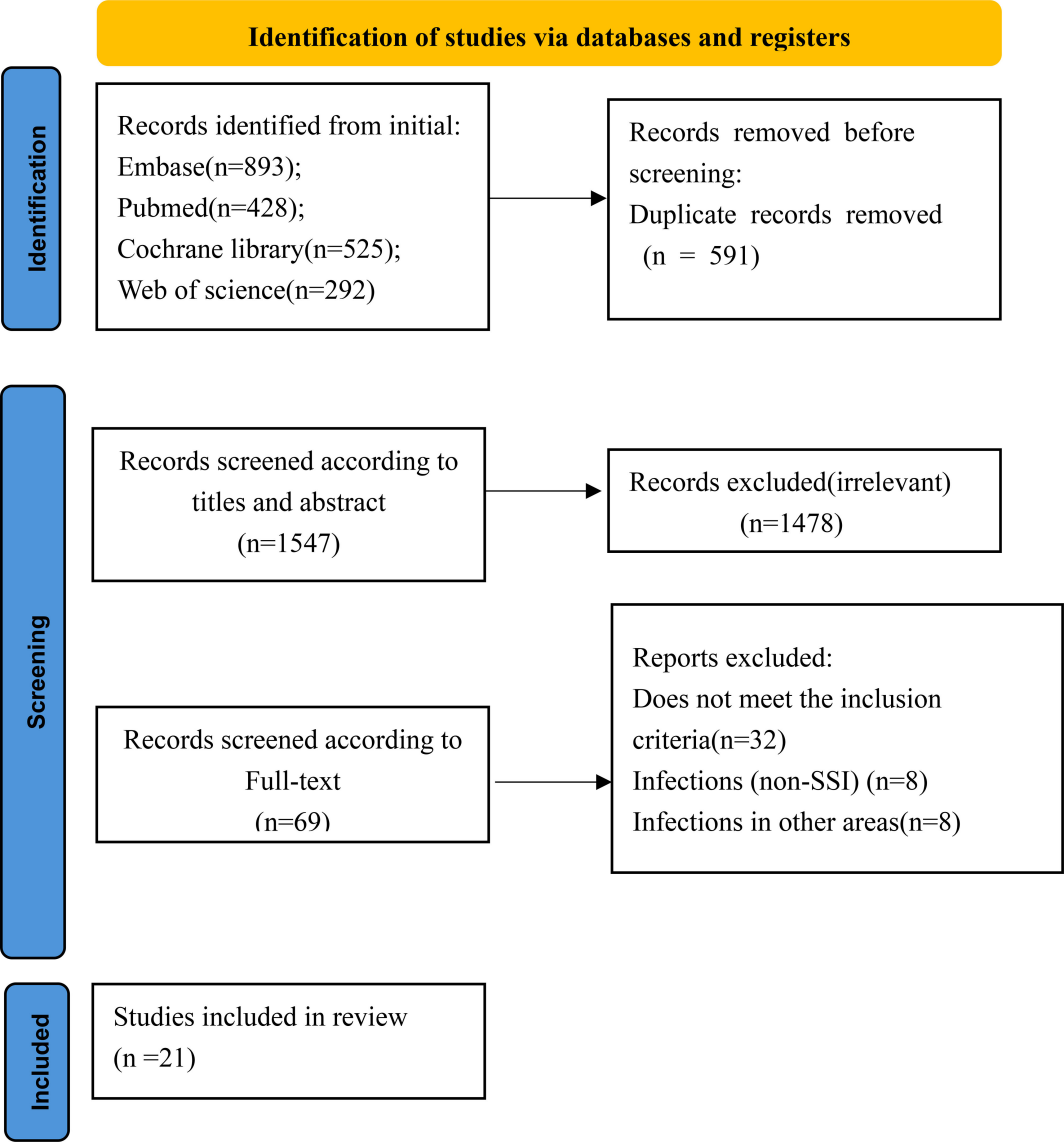
The literature selection process.

The systematic search identified 2,138 records. After removing duplicates and screening titles and abstracts, 69 full-text articles were assessed. Finally, 21 studies met the inclusion criteria and were included in the meta-analysis. The detailed selection process is shown in **Figure 1**.

### 3.2 Study characteristics

A total of 21 studies (17 retrospective cohort studies, 3 case-control studies, and 1 prospective cohort study) (8–28). The main characteristics of these studies are summarized in **Table 1**. The studies were published between 2007 and 2024 and included patients treated from 1977 to 2023. Overall, 1514 SSI events were reported. The definition of SSI across studies varied and encompassed several categories, including: 1) The National Nosocomial Infections Surveillance (NNIS) (29); 2) Centers for Disease Control (CDC) (29); 3) Johnson’s criteria (30); 4) the presence of purulent discharge from the wound, or exudate with signs of local infection and positive cultures.

**Table 1:** The characteristics of the 21 included studies.

Study	Country	Study type	Timing of surgery	Total	SSI	Age	F/M	SSI definitions	Significant factors
Yao, 2017 (19)	Canada	retrospective	1977-2014	365	84	59.2	139/226	CDC, NNIS	age, sex, diabetes, smoking, mandibulectomy, RT
Wang, 2016 (20)	China	retrospective	1995-2014	143	33	71.3 ± 2.7	68/75	CDC, NNIS	BMI, diabetes, ASA classification, operative time, reconstructive surgery
Shi, 2020 (13)	China	retrospective	2005-2016	786	125	58.5	325/461	CDC	diabetes, RT, tracheostomy, oral–neck communication
Shen, 2023 (10)	China	retrospective	2014-2019	168	22	NA	73/95	CDC, NNIS, Johnson’s criteria	age, BMI, ASA classification, diabetes, RT, neck dissection, transtracheal resection; transfusion, operative time, ALB, PLR, NLR
Stao,j, 2011 (25)	Japan	Case–control	2005-2007	66	14	68	26/40	Johnson’s criteria	T-stage, neck dissection, tissue transplantation, oral health care, RT, operative time, denture blood transfusion
Nakamura, 2020 (14)	Japan	retrospective	2009-2017	106	28	58	40/60	CDC	BMI, lower hemoglobins, Lower SMI
Makiguchi, 2019 (15)	Japan	retrospective	2009-2017	88	27	64.8	31/57	CDC	hypoalbuminemia, free flap
Makiguchi, 2019 (16)	Japan	retrospective	2009-2018	122	30	60.4	40/82	CDC	SMI, underweight, anemia
Ma, 2012 (23)	China	retrospective	2004-2010	376	87	NA	193/183	CDC, NNIS	BMI, diabetes, ASA classification, ACE-27, reconstruction, operative time
Liu, 2011 (26)	China	Case–control	1994-2007	306	95	50.3 ± 9.9	10/296	Johnson’s criteria	diabetes, mandibulectomy, blood transfusion
Liu, 2007 (28)	China	retrospective	1995-2003	994	197	51.5 ± 11.7	68/926	Johnson’s criteria	age, sex, diabetes, neck dissection, T-stage, reconstruction, ALB, blood transfusion
Lin, 2012 (24)	China	retrospective	2004-2010	894	186	51.2 ± 10.7	33/861	Johnson’s criteria	age, diabetes, operative time,blood transfusion, gender, neck dissection
Lin, 2018 (17)	China	retrospective	2010-2015	173	67	55.63	9/164	CDC	operative time, mandibulectomy, oro-neck defect
Lee, 2015 (22)	Korea	retrospective	2008-2012	337	88	57	102/235	CDC,NNIS, Johnson’s criteria	Tumor site, RT, tracheostomy, operative time, blood transfusion, Mandibulectomy; flap reconstruction
Karthik, 2021 (12)	India	retrospective	2012-2020	344	67	57 ± 19	120/224	CDC	Mandibulectomy, tracheostomy, flap reconstruction, blood loss, time under anesthesia
Karakida, 2010 (27)	Japan	retrospective	1996-2005	276	112	63	106/170	CDC	ASA classification, duration of surgery
Hiraoka, 2022 (11)	Japan	retrospective	2007-2015	67	28	65	25/42	NNIS	ALB,PLR
Fujii, 2024 (8)	Japan	retrospective	2014-2019	120	45	60	35/85	CDC	SMI, sex, age, ACE-27, PNI
Elfayeg, 2024 (9)	Sudan	prospective	2022-2023	60	26	NA	18/42	CDC	clinical stage, sutures, blood transfusion, duration of hospital stay
Eder-Czembirek, 2016 (21)	Australia	retrospective	2004-2011	85	38	58.4	26/59	CDC,NNIS, Johnson’s criteria	BMI, alcohol, diabetes
Belusic-Gobic, 2018 (18)	Croatia	Case–control	2009-2015	195	115	NA	45/150	Signs of wound infection	gender, smoking, tumor size, T-stage, tracheotomy, mandibular resection, gastrostomy, reconstruction surgery

SSI, surgical site infection; CDC, Centers for Disease Control; NNIS, The National Nosocomial Infections Surveillance; RT, radiotherapy; BMI, Body mass index; ASA, American Society of Anesthesiologists; ALB, serum albumin level; PLR, platelet -to-lymphocyte radio; NLR, neutrophil-to-lymphocyte ratio; T-stage, tumor stage; SMI, skeletal mass index; ACE, adult comorbidity evaluation; NA, No available; PNI, prognostic nutritional index.

**Table 2** summarizes the specific results of literature quality evaluation. According to the Newcastle–Ottawa Scale (NOS), 16 studies were classified as high quality (≥7 points), while 2 studies were of moderate quality (6 points). The remaining studies also met acceptable quality standards.

**Table 2:** Quality evaluation of the included literature.

Study	Score situation			
	Selection	Comparability	Exposure	Overall score
Yao, 2017 (19)	3	1	2	6
Wang, 2016 (20)	3	1	1	5
Shi, 2020 (13)	3	2	2	7
Shen, 2023 (10)	3	1	3	7
Stao,j, 2011 (25)	3	2	3	8
Nakamura, 2020 (14)	3	1	3	7
Makiguchi, 2019 (15)	3	2	2	7
Makiguchi, 2019 (16)	3	2	3	8
Ma, 2012 (23)	3	1	3	7
Liu, 2011 (26)	3	1	3	7
Liu, 2007 (28)	3	1	2	7
Lin, 2012 (24)	3	1	3	7
Lin, 2018 (17)	3	2	2	7
Lee, 2015 (22)	3	1	2	6
Karthik, 2021 (12)	3	2	3	8
Karakida, 2010 (27)	3	1	3	7
Hiraoka, 2022 (11)	2	1	2	5
Fujii, 2024 (8)	3	2	2	7
Elfayeg, 2024 (9)	3	1	1	5
Eder-Czembirek, 2016 (21)	3	2	3	8
Belusic-Gobic, 2018 (18)	3	2	3	8

### 3.3 incidence of SSI

The pooled incidence of SSI was 29% (95% CI: 25%–33%) based on 21 studies (**Table 3**; **Supplementary Figure 1**), with substantial heterogeneity (I² = 92.7%, p < 0.001). Subgroup analyses were conducted based on variables such as sex, tumor location, tumor stage (clinical and TNM stage), reconstructive surgery, and neck dissection. Key findings showed a higher SSI incidence in advanced-stage disease (Stage III/IV: 34%; T3/T4: 42%) compared with early-stage disease (Stage I/II: 21%; T1/T2: 26%).

**Table 3:** The incidence of surgical site infection in oral cancer.

Variable	No. of study	No. of the total patients	No of SSI	I² value	P value	Effects model	SSI incidence rate	P value
Total	21	6071	1514	92.70%	0.000	random	0.29(0.25,0.33)	0.000
Female	18	1391	317	77.20%	0.000	random	0.25(0.20,0.30)	0.000
Male	19	4205	1047	92.40%	0.000	random	0.29(0.24,0,34)	0.000
Tumor site								
tongue	10	1383	306	82.90%	0.000	random	0.25(0.20,0.31)	0.000
Floor of mouth	9	342	111	65.50%	0.003	random	0.33(0.24,0.41)	0.000
Gingiva	8	681	191	92.40%	0.000	random	0.33(0.21,045)	0.000
Buccal	9	1237	291	79.50%	0.000	random	0.30(0.23,0.36)	0.000
Palate	7	140	17	39.90%	0.140	fixed	0.09(0.04,0.13)	0.002
Clinical stage								
I, II	8	1208	227	87.10%	0.000	random	0.21(0.14,0.27)	0.000
III, IV	10	1254	415	88.90%	0.000	random	0.34(0.26,0.42)	0.000
T-stage								
T1+T2	8	145	335	89.60%	0.000	random	0.26(0.19,0.34)	0.000
T3+T4	8	902	339	92.60%	0.000	random	0.42(0.31,0.54)	0.000
N-stage								
N(-)	7	688	247	91.30%	0.000	random	0.37(0.25,0.49)	0.000
N(+)	8	663	245	87.10%	0.000	random	0.39(0.28,0.50)	0.000
Reconstructive method								
free flap	8	921	307	86.00%	0.000	random	0.32(0.24,0.40)	0.000
suture	5	502	39	42.00%	0.142	fixed	0.07(0.05,0.09)	0.000
Local flap	4	725	88	90.60%	0.000	random	0.16(0.06,0.26)	0.002
Neck dissection								
NO	7	2252	1646	95.70%	0.000	random	0.67(0.58,0.77)	0.000
Bilateral	7	183	103	88.10%	0.000	random	0.48(0.28,0.69)	0.000
Unilateral	5	538	206	98.40%	0.000	random	0.35(0.10,0.60)	0.006

Free flap reconstruction was associated with a higher SSI rate (32%) than suture closure (7%) or local flaps (16%). Extensive neck dissection (bilateral: 48%) was associated with a higher risk compared with unilateral (35%) or no dissection. The findings from a comprehensive analysis of subgroup analyses on SSI incidence are presented in **Table 3**, revealing significant heterogeneity ranging from moderate to high levels.

### 3.4 Risk factors associated with SSI

**Table 4** provides a concise summary of the key findings from the meta-analysis on risk factors, while **Supplementary Figure 2** visually presents the forest plot. The results of the meta-analysis demonstrated significant associations between specific factors and SSI in individuals diagnosed with oral cancer (p ≤ 0.05): radiotherapy, mandibulectomy, diabetes, American Society of Anesthesiologists (ASA) classification, operative time, reconstructive surgery, transfusion, neck dissection, and serum albumin (ALB). No statistically significant association was found between SSI and the following risk factors: male sex, BMI, Tracheostomy, skeletal muscle index (SMI). Operative time (categorical ≥6 h) was associated with increased SSI risk (OR = 4.17, 95% CI: 2.17–7.99). Three studies reported operative time as a continuous variable with inconsistent units (e.g., minutes or hours); therefore, these results were not pooled. Among these, two studies suggested a positive association between longer operative time and SSI, while one study showed no statistically significant association.

**Table 4:** Risk factors of surgical site infection in patients with oral cancer.

Factors	NO. of study	I² value	P value	Effects model	OR (95%CI)	P-value	Egger’s test p value
Male sex	5	69.30%	0.011	random	1.44(0.68,3.05)	0.339	0.138
Radiotherapy	5	34.40%	0.192	fixed	1.81(1.16,2.85)	0.010	0.157
Mandibulectomy	5	36.20%	0.180	fixed	2.82(2.00,3.98)	0.000	0.024
Diabetes	6	77.90%	0.000	random	3.18(1.87,5.40)	0.000	0.146
BMI	3	71.20%	0.031	random	2.13(0.93,4.87)	0.074	0.051
ASA classification	4	34.60%	0.205	fixed	1.40(1.23,1.60)	0.000	0.029
ACE-27	3	71.20%	0.031	random	1.01(0.60,1.71	0.974	0.078
Operative time (≥6 h, categorical)	5	85.9%	0.000	random	4.17 (2.17–7.99)	0.000	0.455
Reconstructive surgery	5	82.50%	0.000	random	4.10(2.10,7.99)	0.000	0.100
Tracheostomy	3	64.20%	0.061	random	1.63(0.61,4.32)	0.326	0.765
Transfusion	6	71.00%	0.004	random	3.18(1.56,6.48)	0.001	0.447
Neck dissection	3	0.00%	0.976	fixed	1.93(1.09,3.40)	0.023	0.784
Lower SMI	2	66.10%	0.086	random	1.99(0.79,4.98)	0.142	1.000
Albumin	2	92.10%	0.000	random	2.20(1.07,4.53)	0.032	0.079

### 3.5 Sensitivity analysis and publication bias

Sensitivity analysis was performed by sequentially excluding individual studies for variables with high heterogeneity (e.g., diabetes, operative time, reconstructive method, transfusion, albumin). The pooled effect size did not change substantially, indicating the results were robust. Publication bias tests were conducted for the risk factors included in ≥ 2 studies. The studies investigating mandibulectomy, ASA classification, and operative time risk factors exhibited significant publication bias according to the Egger’ s test (**Table 4**).

## 4 Discussion

In this meta-analysis, 1,514 cases of SSI were identified among 6,071 patients, corresponding to a crude incidence of 25.5%. The pooled incidence of SSI was 29% (95% CI: 25%–33%); however, substantial heterogeneity was observed (I² = 92.7%). This suggests considerable variability across studies in terms of patient populations, surgical procedures, and clinical settings. Therefore, this estimate should be interpreted as an average across heterogeneous contexts rather than a universally applicable incidence rate. Future studies with more standardized definitions and designs are needed to provide more precise estimates.

Although several factors identified in this study have been previously reported, our meta-analysis provides an updated and more comprehensive synthesis of specific to oral cancer surgery. By including a larger number of studies and patients, as well as more recent data, this study allows for more precise estimation of effect sizes. Furthermore, our analysis highlights methodological variability across studies, including differences in definitions and adjustment strategies, thereby refining and contextualizing current evidence to improve risk assessment and clinical decision-making.

Several methodological considerations should be noted. First, all included effect estimates were derived from multivariable analyses; however, the specific confounders adjusted for varied across studies. Therefore, the pooled results should be interpreted as associations based on heterogeneous multivariable models rather than strictly comparable independent effects. Second, both cohort and case-control studies were included. While these designs differ, the use of odds ratios enabled quantitative synthesis across study types. Third, several variables identified in this study—including operative time, reconstructive procedures, and extent of surgery (e.g., mandibulectomy)—are likely interrelated and reflect overall surgical complexity. Due to the limitations of aggregate-level meta-analysis, collinearity could not be assessed. Thus, these findings should not be interpreted as independent effects but rather as overlapping indicators of surgical complexity. In this study, all eligible studies were included regardless of their quality scores to ensure a comprehensive synthesis of available evidence. While restricting analysis to high-quality studies could potentially strengthen the robustness of the findings, this approach may also reduce statistical power and exclude relevant data. Future research with larger numbers of high-quality studies is needed to further validate the findings.

Clinically, multiple factors were found to be associated with increased SSI risk, including radiotherapy, neck dissection, reconstructive surgery, mandibulectomy, prolonged operative time, diabetes, ASA classification, hypoalbuminemia, and blood transfusion. These findings are generally consistent with those reported by Chen et al., although our study incorporates more recent data and a broader range of variables, thereby complementing existing evidence.

As an adjunctive therapy for oral cancer, radiotherapy employs high-energy rays to eradicate cancer cells but also induces damage to healthy cells (31). These radiation-induced side effects—including DNA mutation, microvascular impairment, and soft tissue fibrosis—impede collagen deposition and angiogenesis during wound healing, with relatively prolonged duration. Previous studies have shown radiotherapy, for these reasons, decelerates wound healing and elevates the risk of postoperative wound infection (13). However, the definition of radiotherapy varied across studies (preoperative, postoperative, or unspecified prior radiotherapy), which may influence its association with SSI. Therefore, these results should be interpreted with caution.

Oral cancer metastasis to regional lymph nodes primarily occurs via lymphatic vessels rather than hematogenous spread. Thus, comprehensive neck management is imperative in treating most oral cancer patients (32), with neck dissection widely employed. During oral cancer surgery, oral indigenous bacteria tend to migrate to neck surgical sites via newly established communication pathways.

Surgical factors also play a central role in SSI development. Reconstructive procedures, particularly free flap reconstruction, are associated with increased surgical complexity, prolonged operative time, and larger wound surfaces, all of which increase susceptibility to infection (33) (9, 34). Similarly, mandibulectomy and extensive neck dissection are often performed in advanced disease and may further increase infection risk due to larger defects and compromised tissue conditions (18, 35). However, there is no consensus on the etiology of SSI following mandibulectomy. Karthik et al. hypothesized that it impacts mucosal barrier integrity and bacterial ecological balance, while potential mechanisms include complex defects from aggressive malignancies leading to flap shrinkage, creating internal dead cavities conducive to bacterial overgrowth (17). Since mandibulectomy is frequently associated with advanced cancer, compromised oral hygiene from prolonged cancer history should be considered (36). Extensive wounds, unhealed mandibular inflammation, and implants can further increase infection risk (37, 38).

The duration of surgery is influenced by factors such as the severity of the disease, the complexity of the procedure, and the need for reconstruction. The longer the duration of the operation, the greater the complexity and destructiveness involved, thereby increasing the vulnerability of the wound to microbial exposure and also indicating a higher likelihood of SSI occurrence (39). however, in this study it was reported inconsistently across studies (categorical vs. continuous with different units), and continuous estimates were not pooled. Therefore, its association should be interpreted cautiously.

Patient-related factors are also important. Diabetes mellitus impedes wound healing through microangiopathy and immunosuppression, necessitating meticulous glycemic control (23, 40). The ASA classification, a valuable tool for stratifying patient risk as it incorporates numerous comorbidities for postoperative adverse events (41). It evaluates pre-anesthesia physical state and surgical vulnerability, with higher scores indicating diminished physical condition and heightened surgical risk. In this study’s analysis, higher ASA classifications showed a positive correlation with an increased likelihood of SSI occurrence. Notably, hypoalbuminemia increases the risk of SSI through critical mechanisms: it diminishes collagen synthesis and impairs granulation tissue formation, delaying wound healing; Meanwhile, it weakens the innate immune response, compromising the host’s defense against pathogens (42). Moreover, reduced plasma colloid osmotic pressure due to hypoalbuminemia induces tissue edema, with interstitial fluid infiltration into wounds creating a conducive environment for bacterial growth (43). Clinically, mitigating hypoalbuminemia is pivotal for reducing SSI risk.

For oral cancer surgery, blood transfusion is often necessary due to extensive tissue resection and major reconstructive requirements. Blood transfusion may increase infection susceptibility by suppressing the recipient’s immune system via transfusion-related immune regulation (TRIM)—a phenomenon where transfusions impair host immune surveillance, though white blood cells in donated blood may contribute to this immunosuppression, they are not the sole cause (9, 44). Oral cancer surgery itself already suppresses the patient’s immune system, and transfusion further exacerbates this immunosuppression; combined with the presence of surgical wounds, this dual effect elevates the risk of SSI (45). However, withholding necessary transfusions to prevent SSI is not recommended, especially in emergencies.

Perioperative antibiotic prophylaxis is a cornerstone of SSI prevention in clean-contaminated surgery (46). However, our meta-analysis could not quantitatively assess its impact due to a substantial lack of consistent reporting across the included literature. Only three studies described their antibiotic protocols with any specificity (9, 23, 27). The absence of standardized reporting on antibiotic choice, timing, duration, and compliance precludes any definitive conclusion regarding the adequacy of prophylaxis in the pooled cohort. Most critically, no study included antibiotic prophylaxis as an independent variable in multivariate analyses. Future prospective studies must prioritize the explicit documentation and analysis of antibiotic protocols to isolate their effect from other surgical and patient-related risk factors and to establish evidence-based guidelines tailored to the high-risk population of oral cancer patients.

Preventive strategies for SSI, particularly perioperative antibiotic prophylaxis, are of significant clinical importance. However, antibiotic protocols were inconsistently reported across the included studies and were not analyzed as independent variables, limiting quantitative evaluation. Future research should prioritize standardized reporting and evaluation of antibiotic regimens to support evidence-based antimicrobial stewardship.

Our analysis is predominantly composed of studies originating from East Asia, particularly China and Japan. This geographical distribution, although not intentionally targeted, reflects several significant underlying factors. Primarily, it corresponds to the global epidemiological patterns of oral cavity cancers, which exhibit a notably higher incidence in Asian countries compared to Western regions (47). Consequently, surgical volume and related clinical research output are naturally greater in these high-incidence regions. In contrast, in North America and Europe, the epidemiology of head and neck cancers is increasingly defined by HPV-associated oropharyngeal cancers—diseases that involve distinct anatomical locations, surgical techniques, and potentially different risks for SSI. While this regional focus may limit the generalizability of our pooled estimates to settings with differing clinical practices and demographic profiles, it concurrently offers concentrated and highly pertinent evidence for regions most heavily affected by oral cavity cancers. Therefore, our findings should be primarily interpreted as applicable to contexts similar to those represented in the analysis. Future multinational research efforts are needed to confirm these findings across diverse healthcare systems and to investigate region-specific risk factors.

Several limitations should be acknowledged. First, the observational design of included studies introduces potential bias. Second, substantial heterogeneity was observed, and although subgroup analyses were performed, sources of heterogeneity could not be fully explained. Third, variability in definitions and reporting of risk factors limited comparability across studies. Finally, the lack of standardized reporting on antibiotic use represents an important unmeasured confounder. Despite these limitations, this study provides a comprehensive and updated synthesis of evidence on SSI in oral cancer surgery and offers clinically relevant insights for risk stratification and perioperative management.

## 5 Conclusions

This meta-analysis identified several factors associated with an increased risk of surgical site infection in patients with oral cancer, including radiotherapy, neck dissection, reconstructive surgery, mandibulectomy, prolonged operative time, diabetes, hypoalbuminemia, blood transfusion, and higher ASA classification. These findings may assist in identifying high-risk patients and optimizing perioperative management strategies. However, the results should be interpreted with caution due to substantial heterogeneity and potential collinearity among variables. Future studies with standardized definitions, consistent reporting, and more rigorous analytical approaches are needed to further strengthen the evidence.

## Data Availability

The original contributions presented in the study are included in the article/**Supplementary Material**. Further inquiries can be directed to the corresponding author.
