# Supplementary material for: Incidence and influencing factors of surgical site infection in patients with oral cancer: a systematic review and meta-analysis
Source: Front Oncol. 2026 May 8;16:1796067. doi: 10.3389/fonc.2026.1796067 (PMC13193841; doi:10.3389/fonc.2026.1796067)
Supplement: Supplementary file 1 [file Table1.docx]

A total


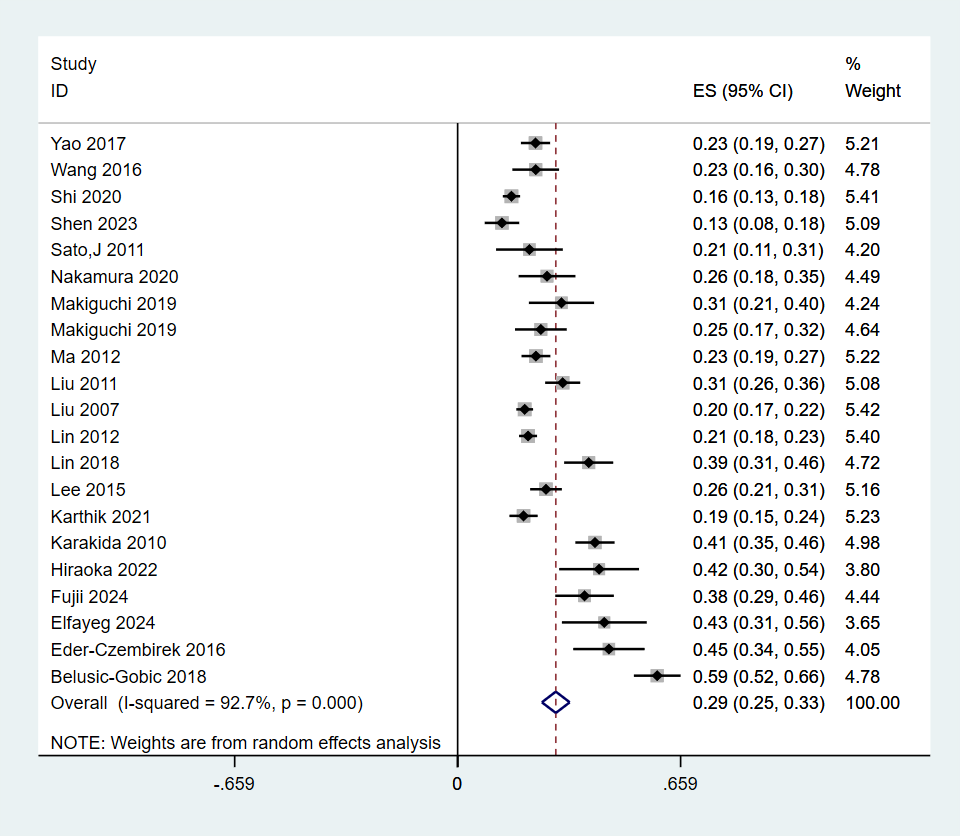


B female


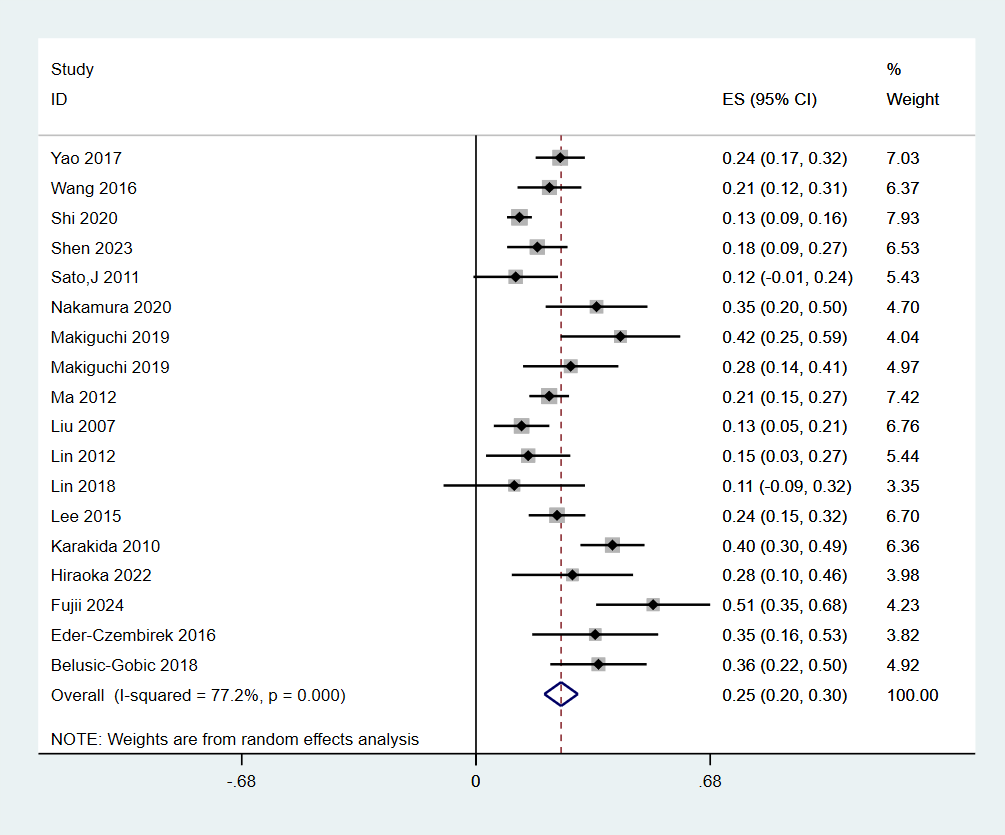


C male


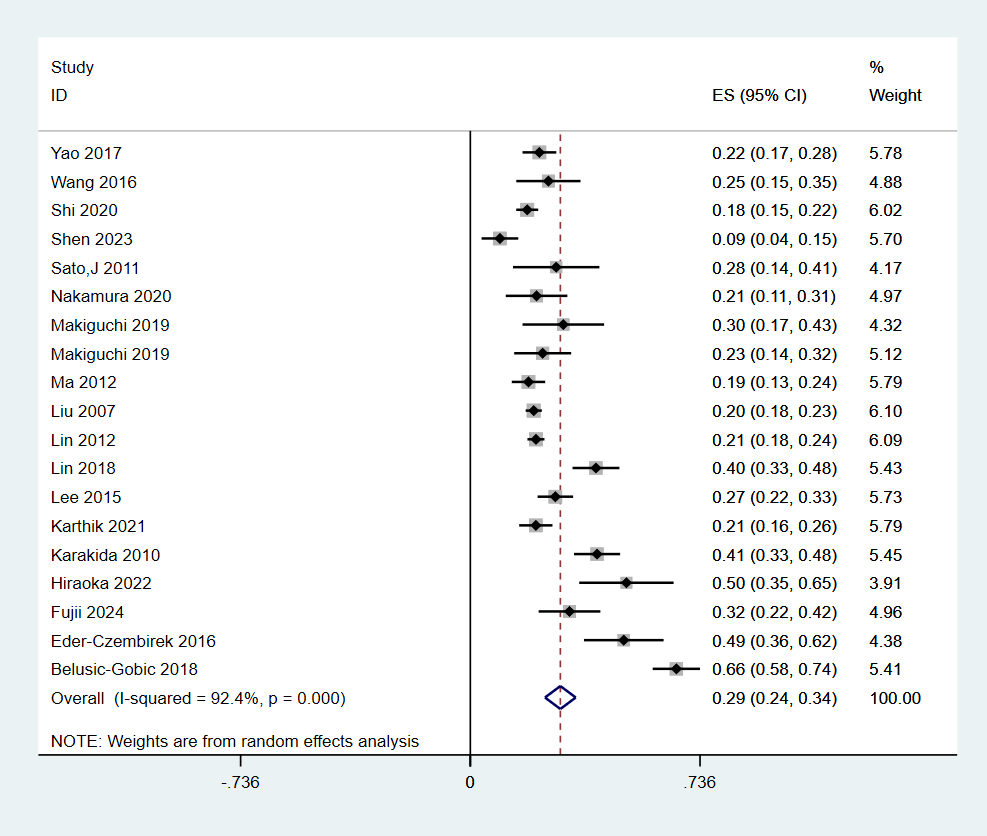


D tongue


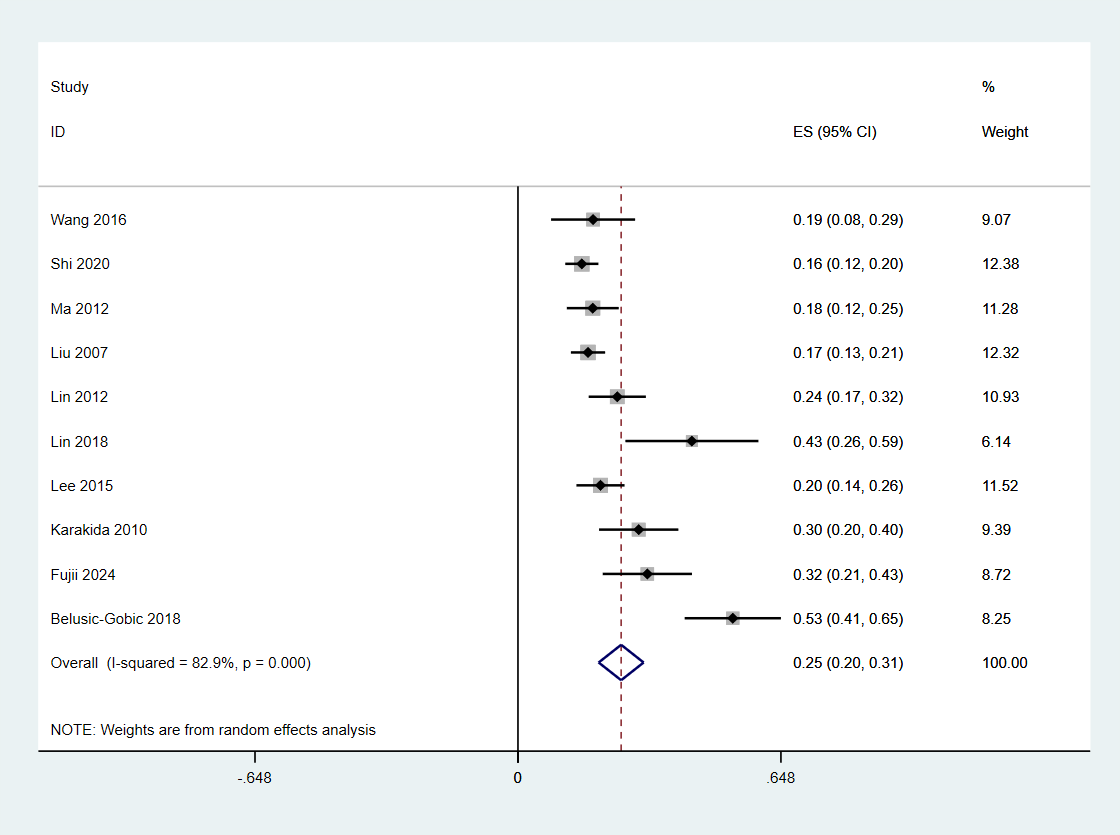


E floor of mouth


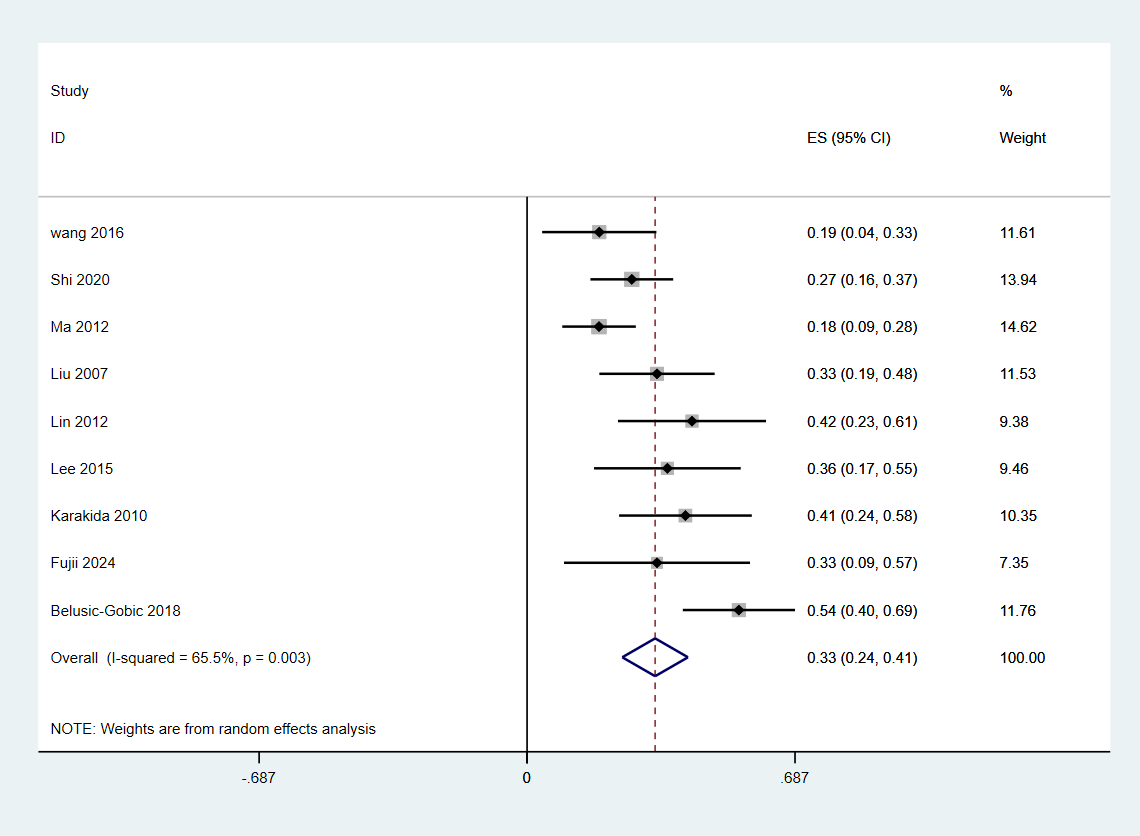


F Gingiva


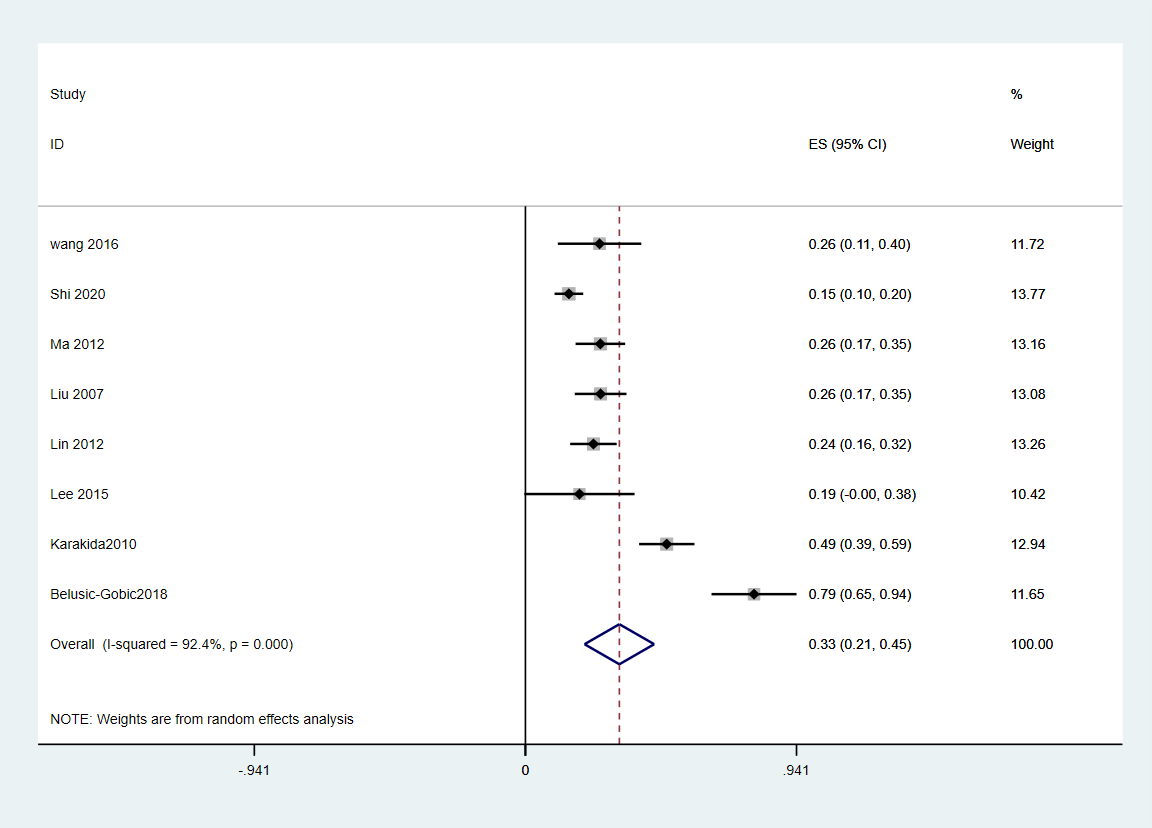


G buccal


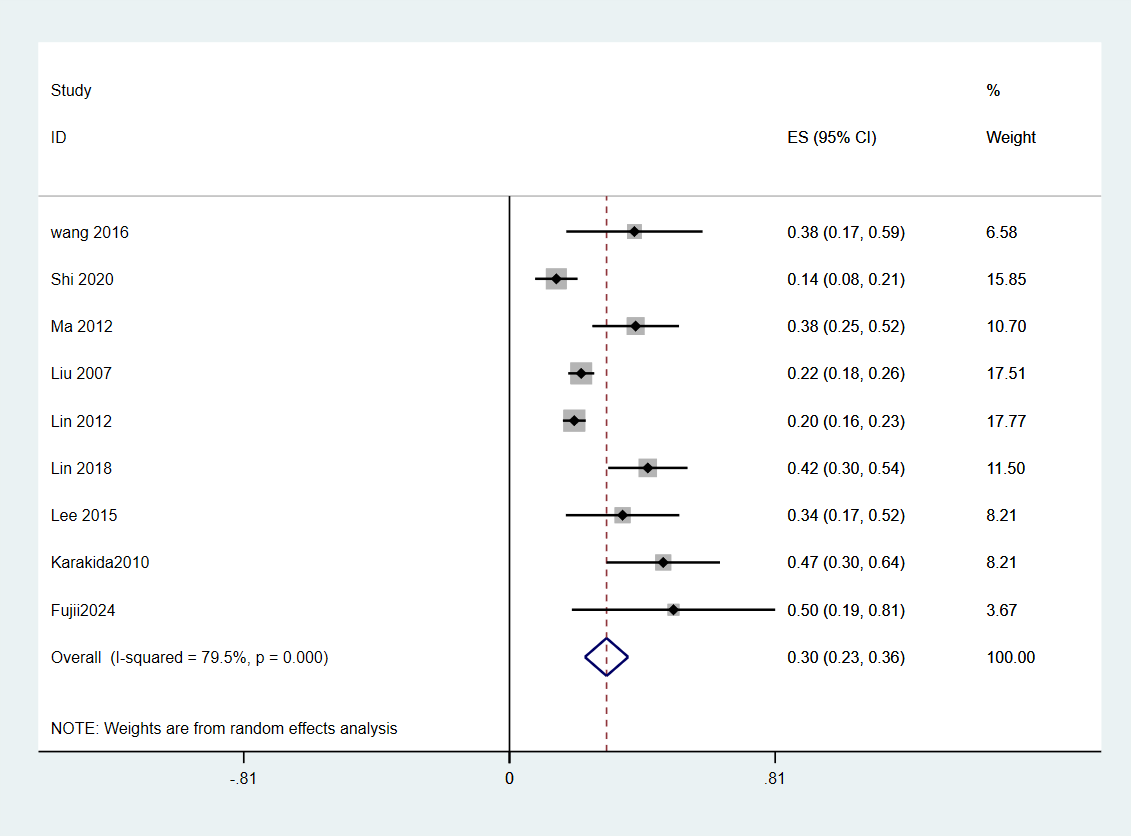


H palate


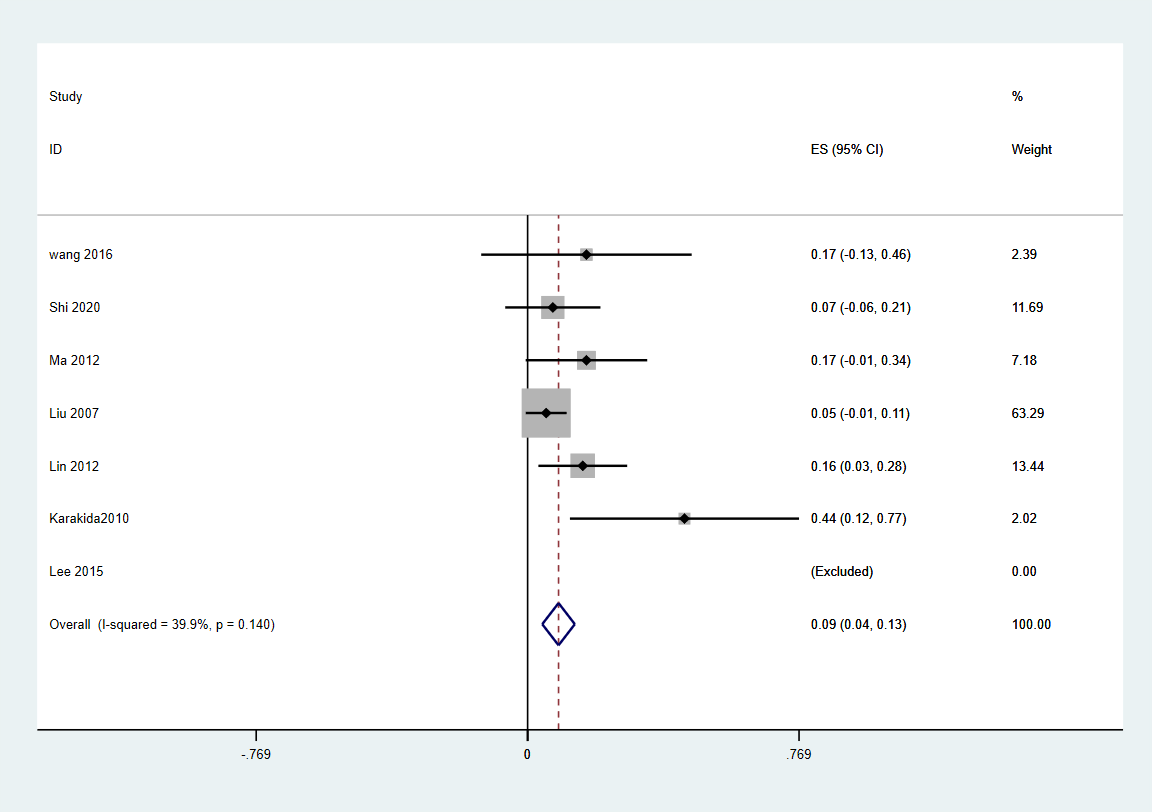


I clinical stage Ⅰ - Ⅱ


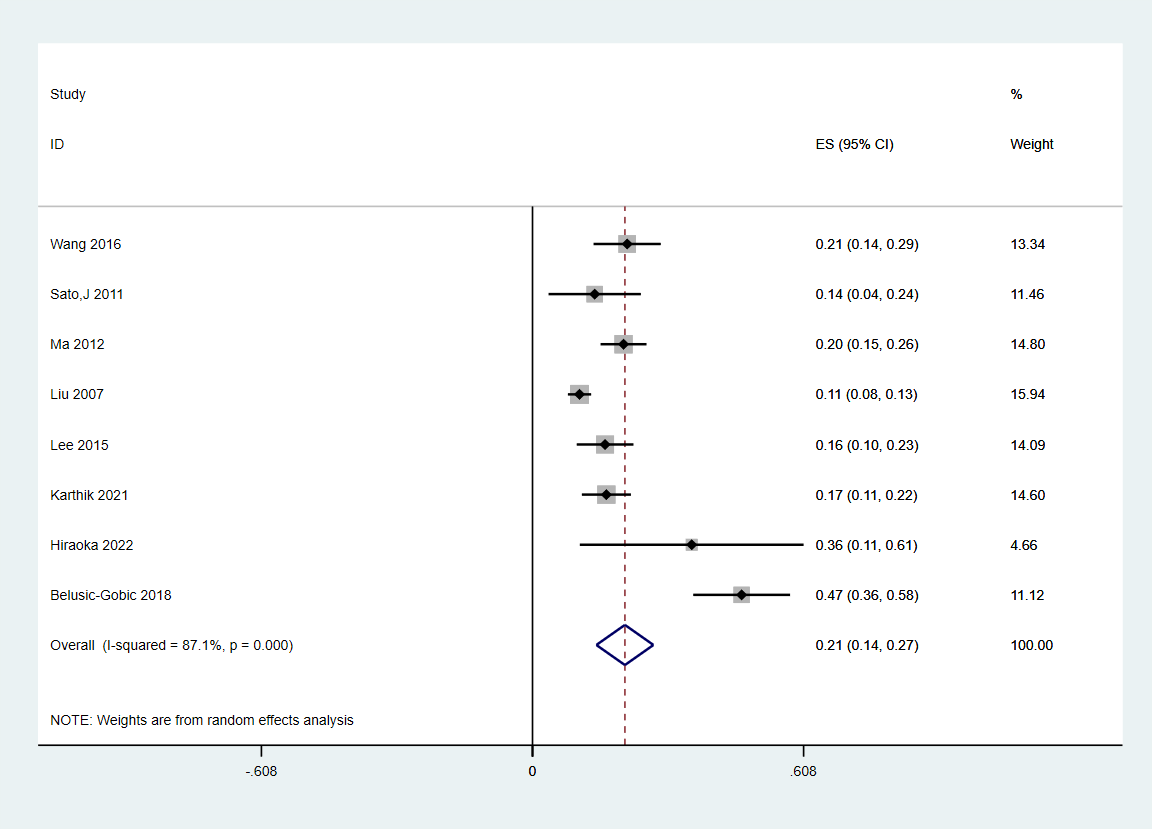


J clinical stage Ⅲ - Ⅳ


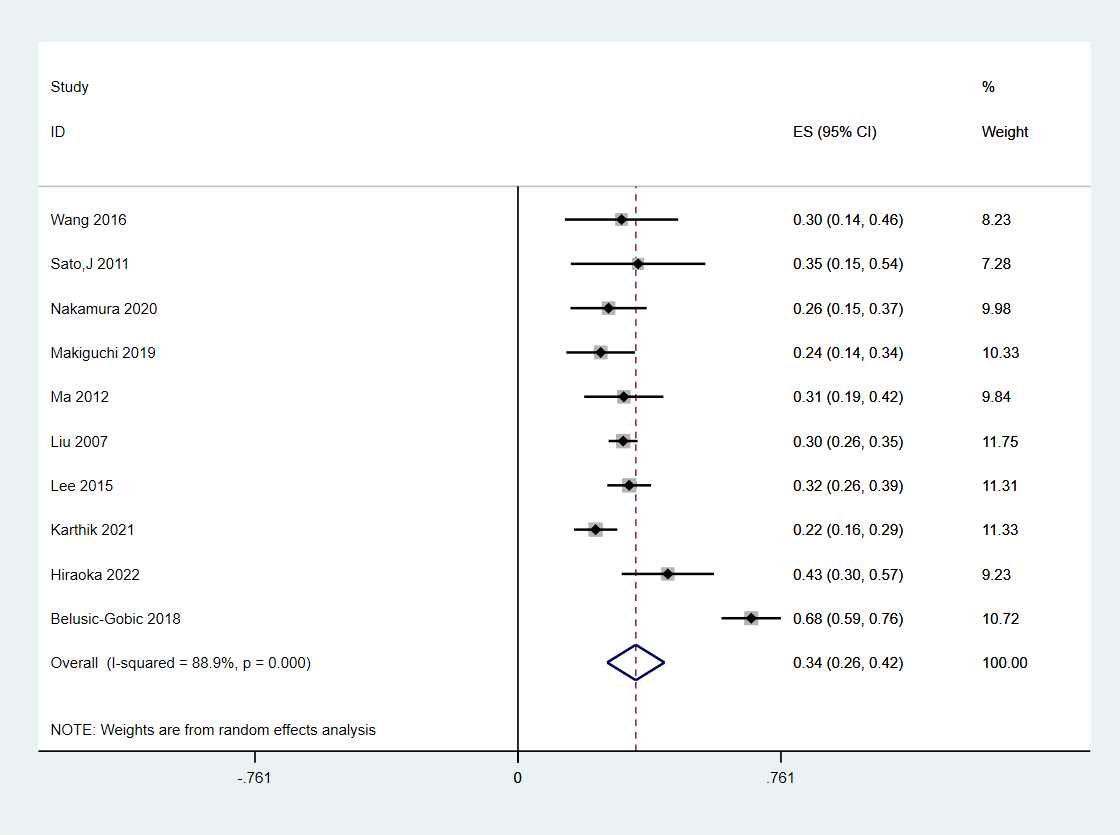


K T1 - T2 stage


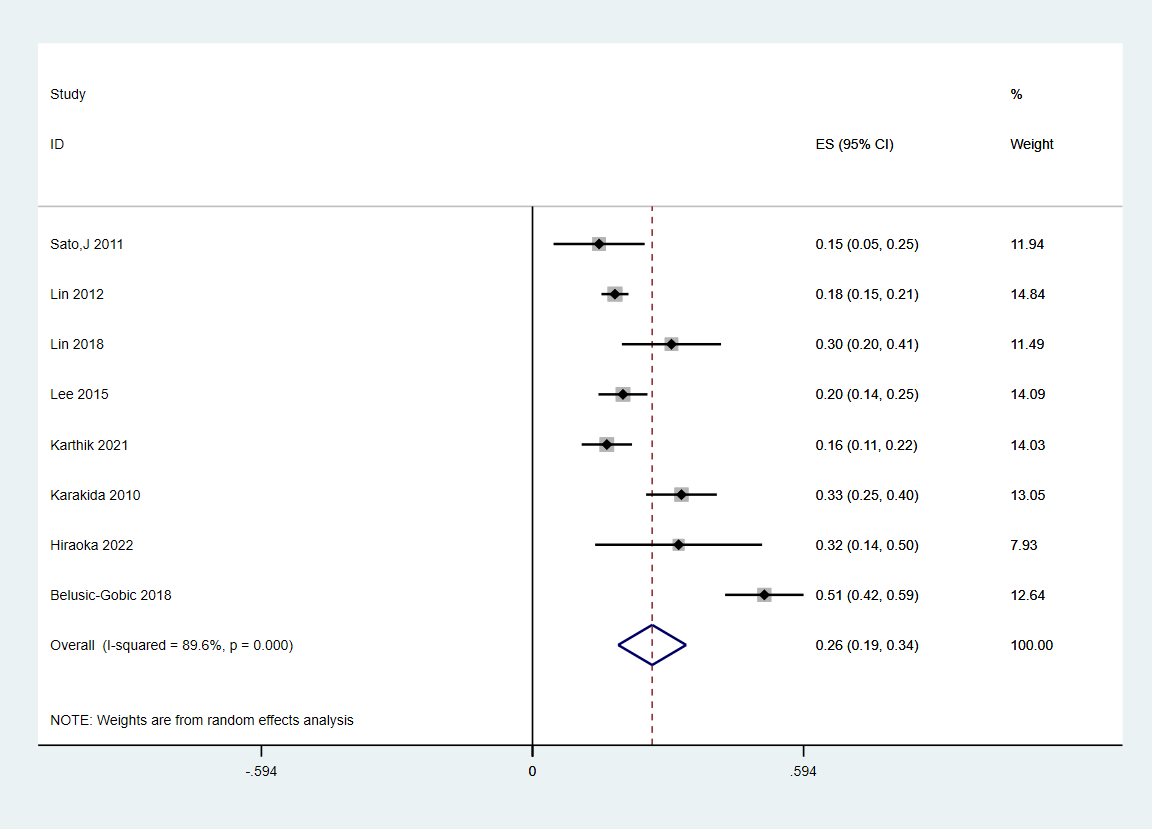


L T3 - T4 stage


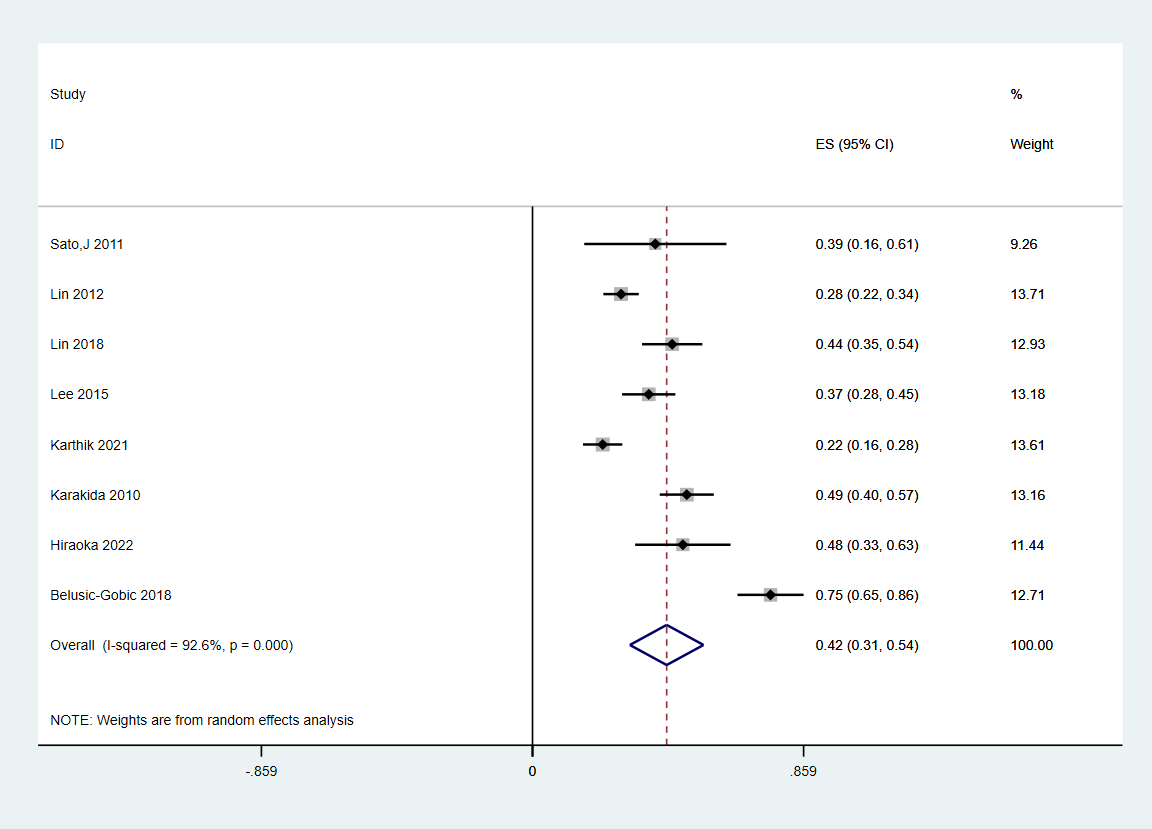


M N(-)


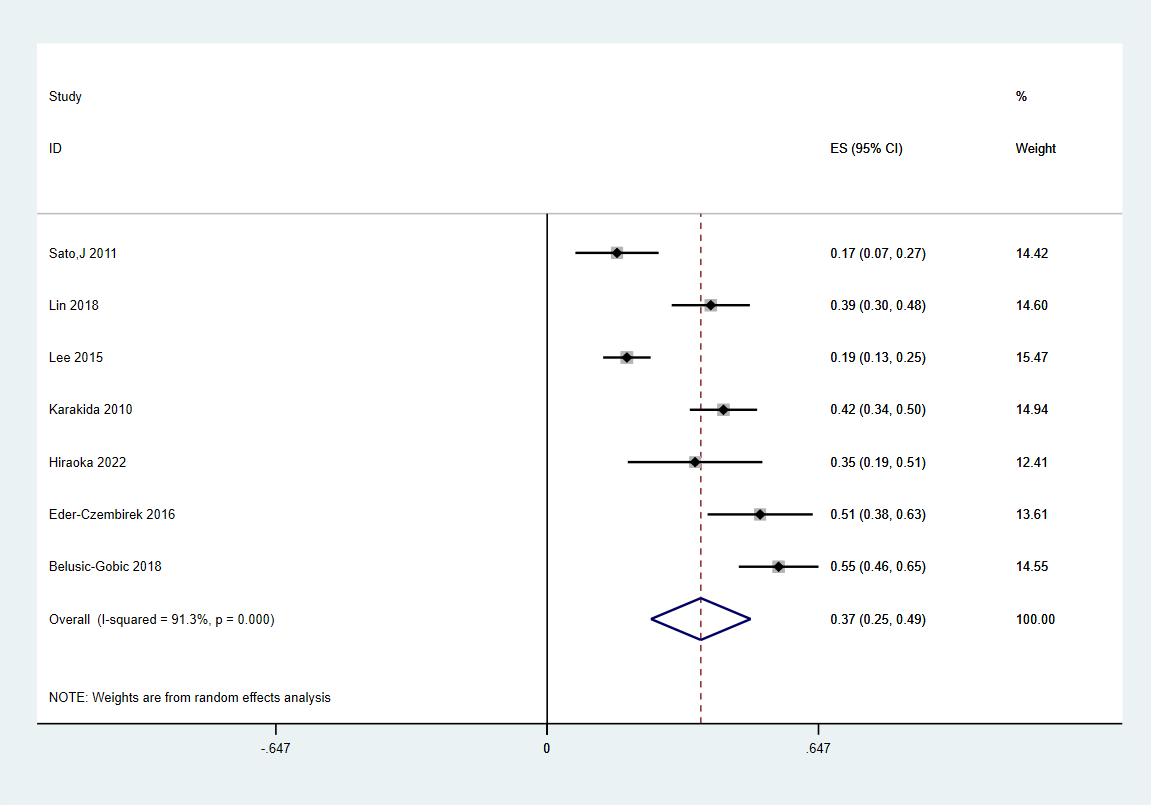


N N(+)


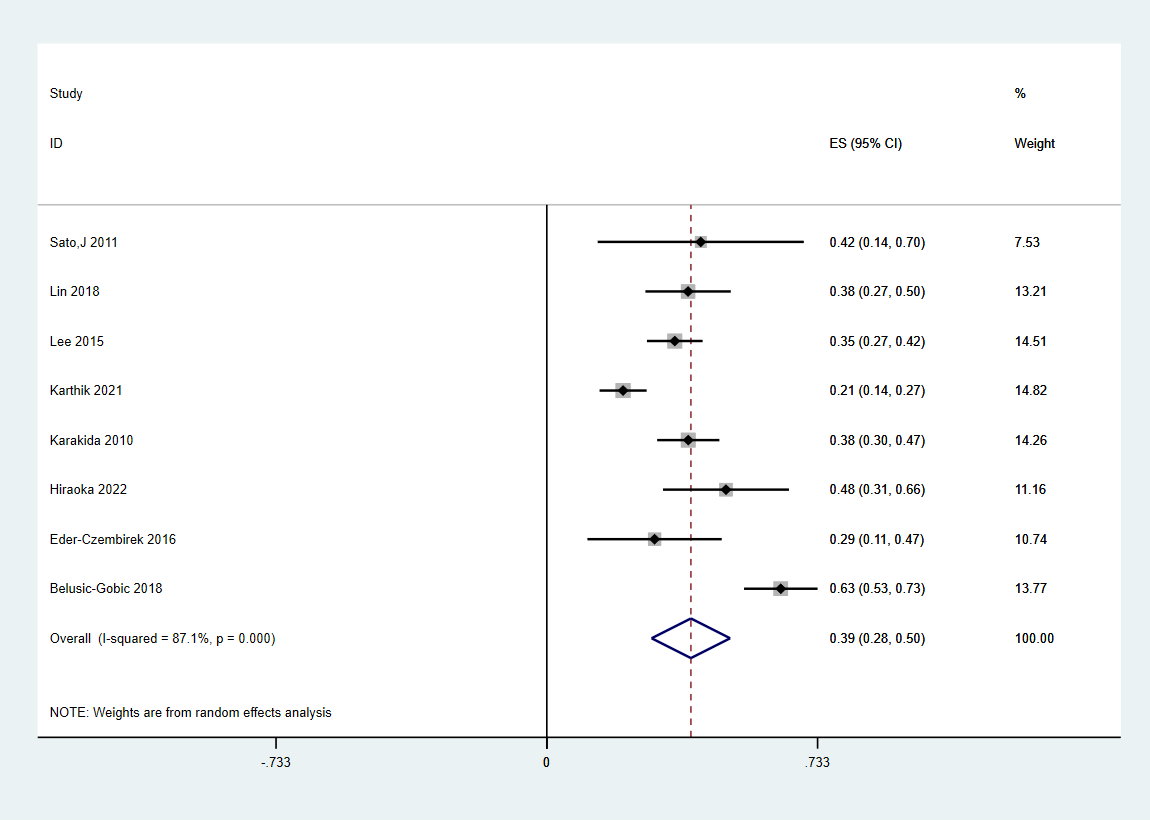


O free flap


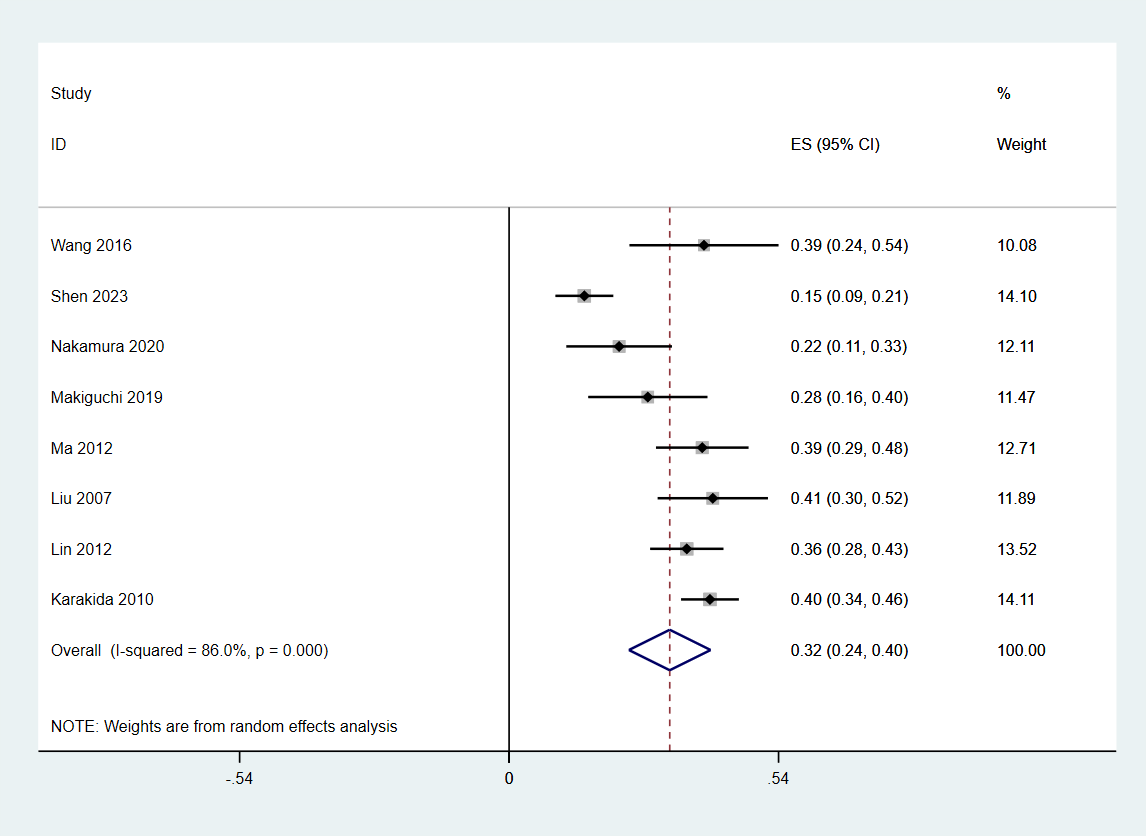


P suture


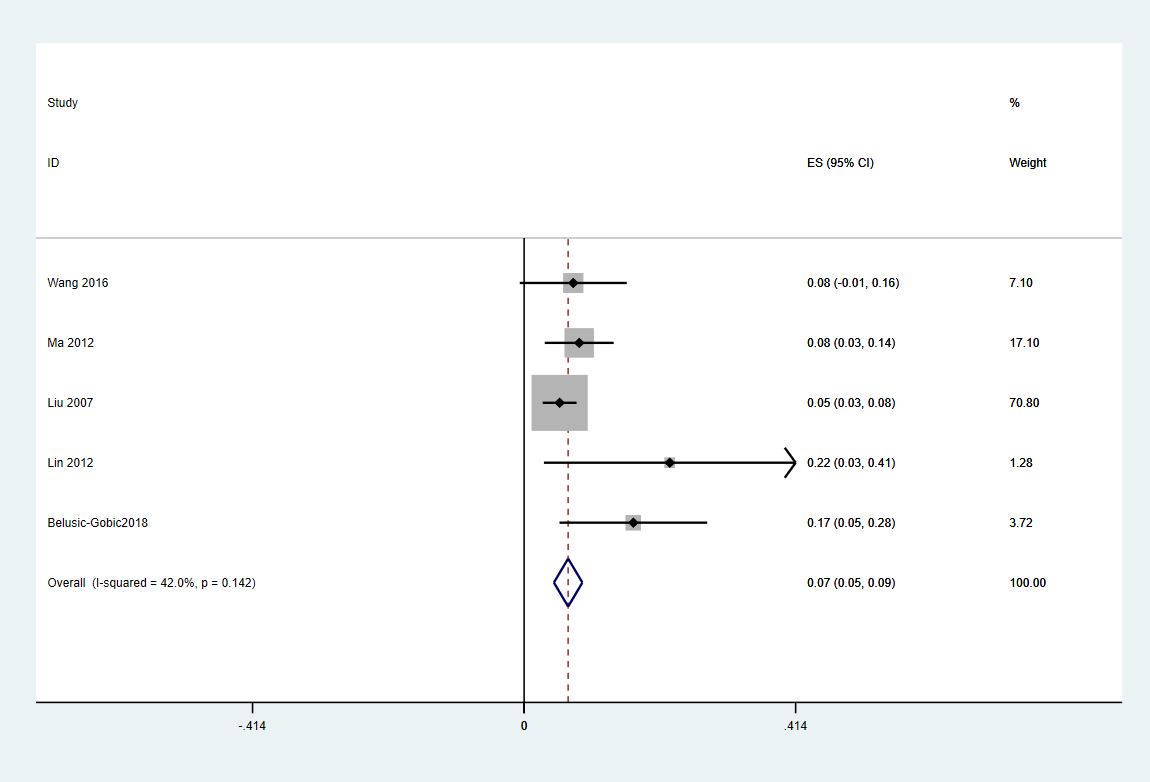


Q Local flap


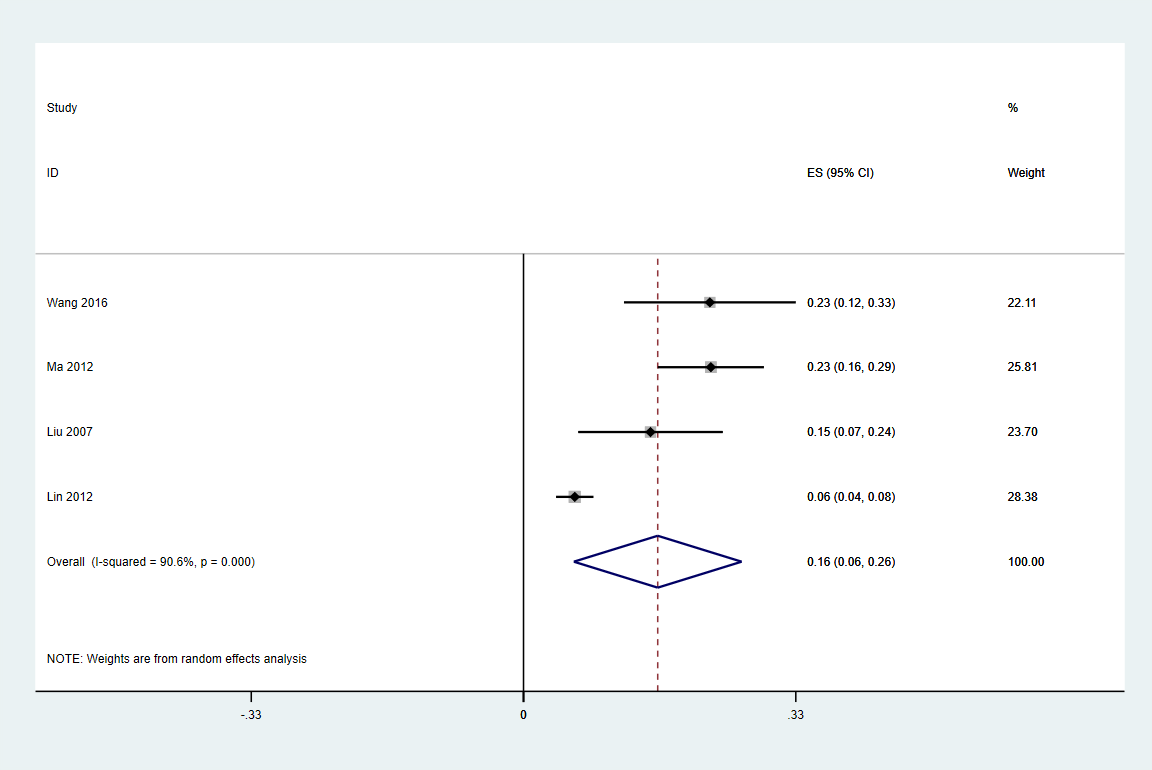


R Neck dissection(NO)


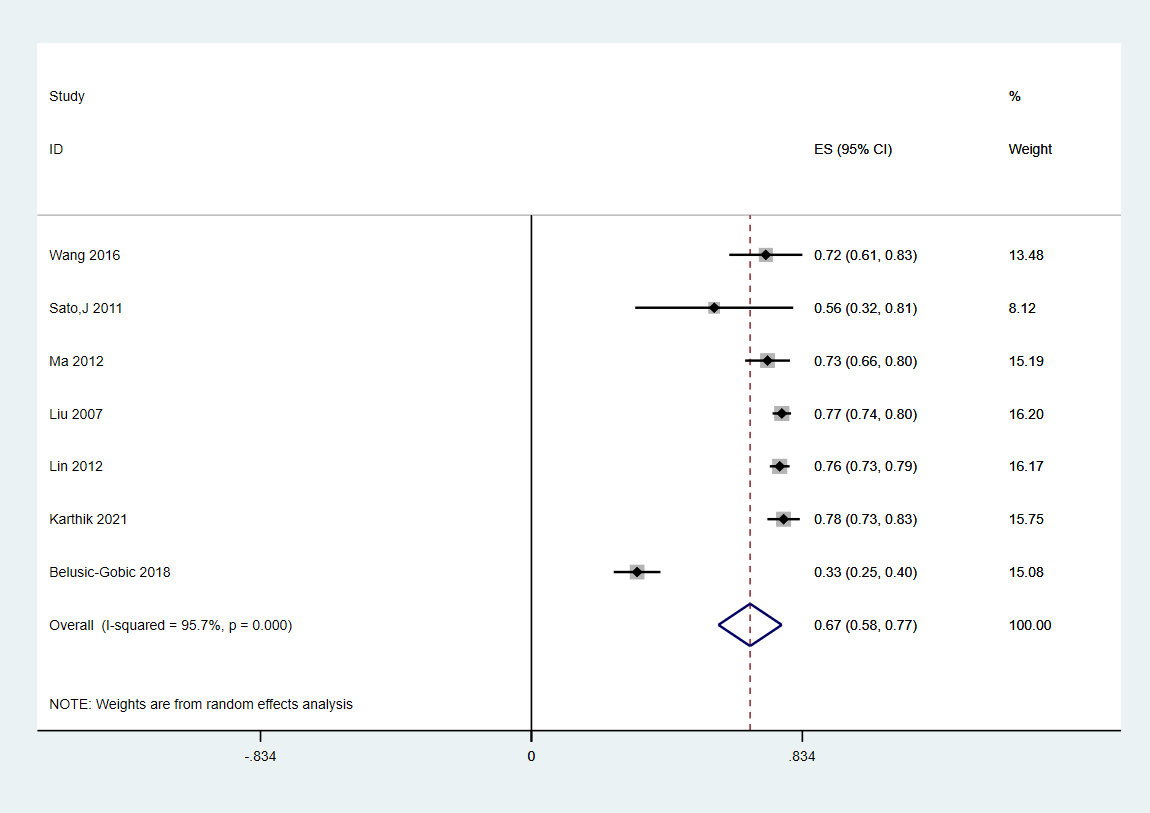


S Neck dissection(Bilateral)


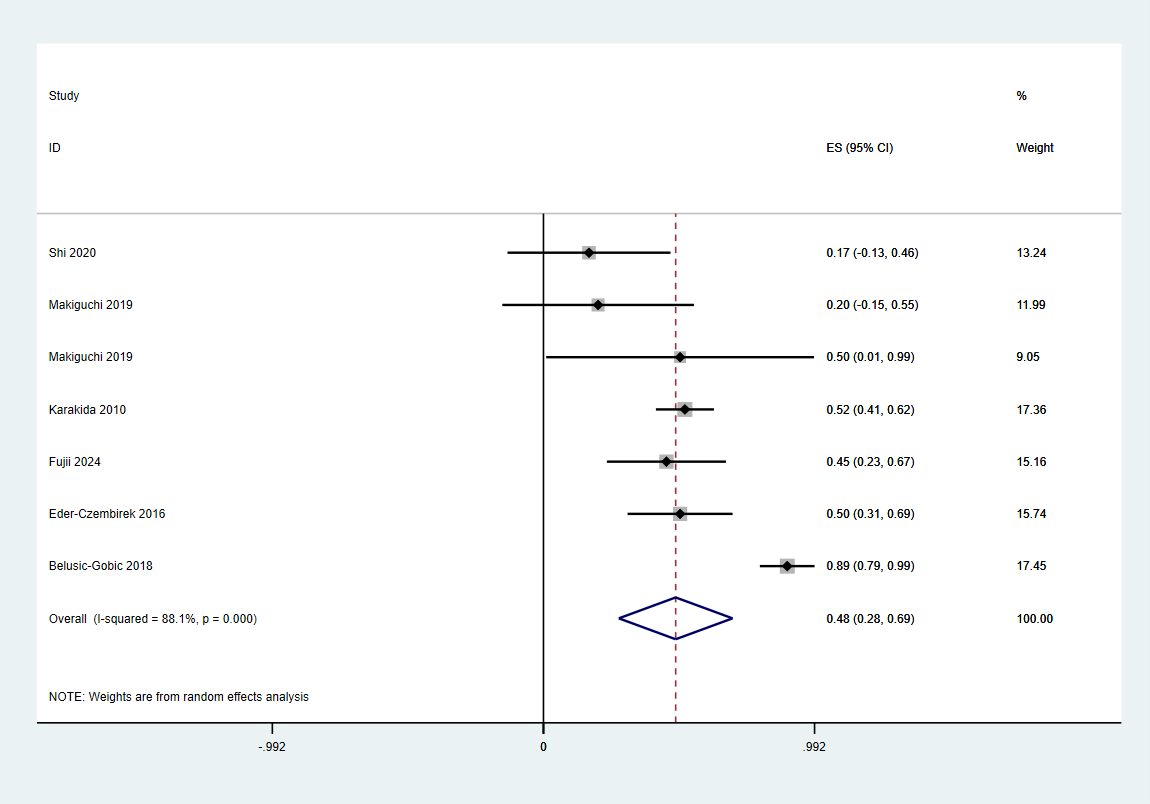


T Neck dissection(Unilateral)


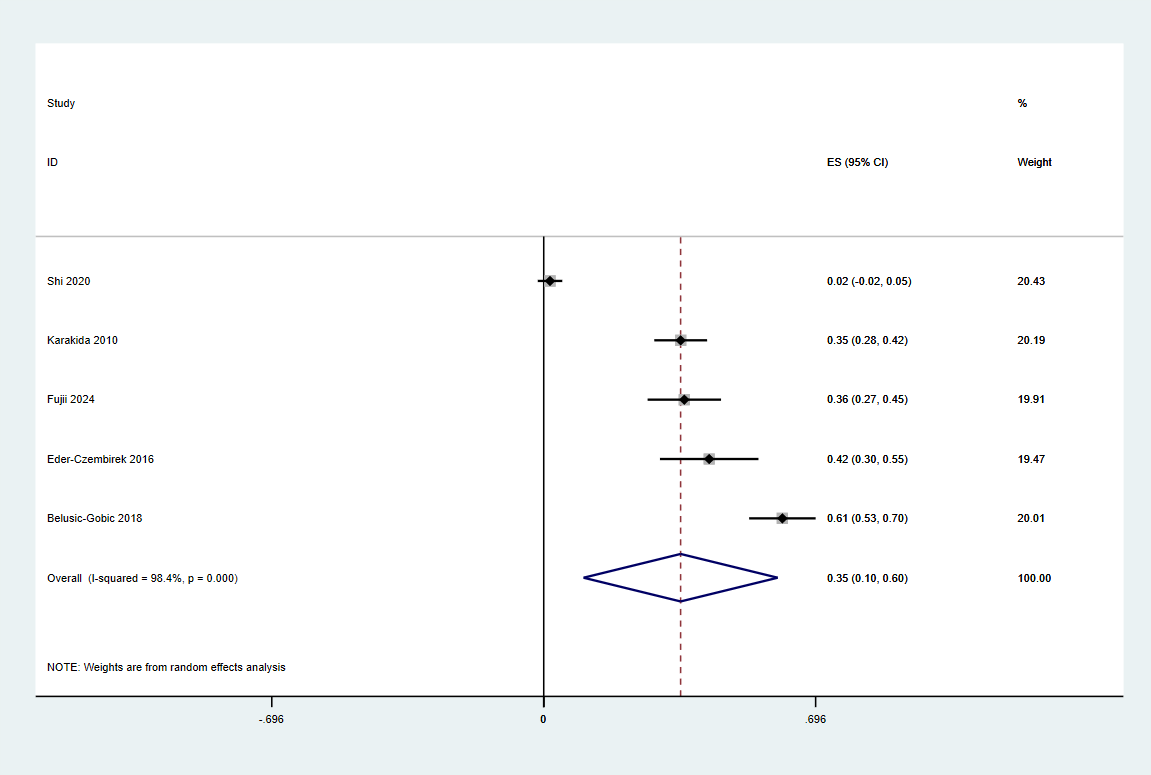


FIGURE S1 Forest plot of Incidence: A total, B female, C male, D tongue, E floor of mouth, F gingiva, G buccal, H palate, I Clinical stage Ⅰ、Ⅱ, J Clinical stage Ⅲ、Ⅳ, K T1、T2 stage, L T3、T4 stage, M N(-), N N(+), O free flap, P suture, Q local flap, R Neck dissection(NO), S Neck dissection(Bilateral), T Neck dissection(Unilateral)

A male


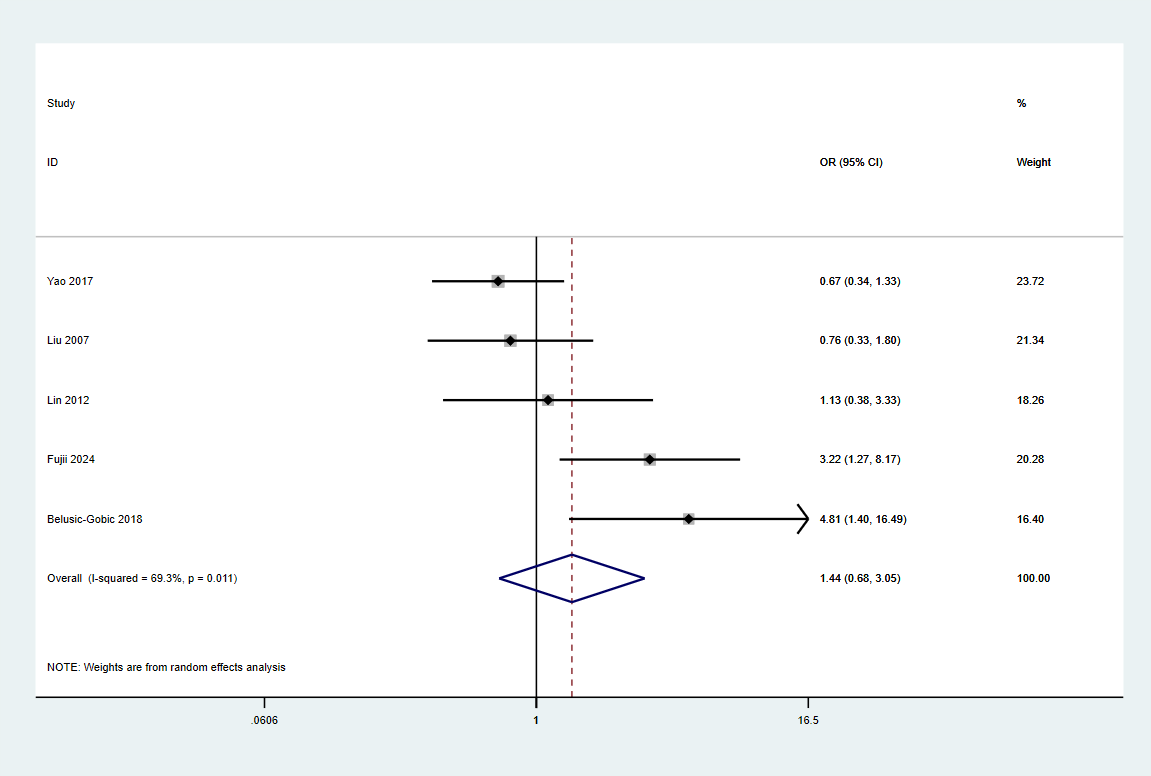


B radiotherapy


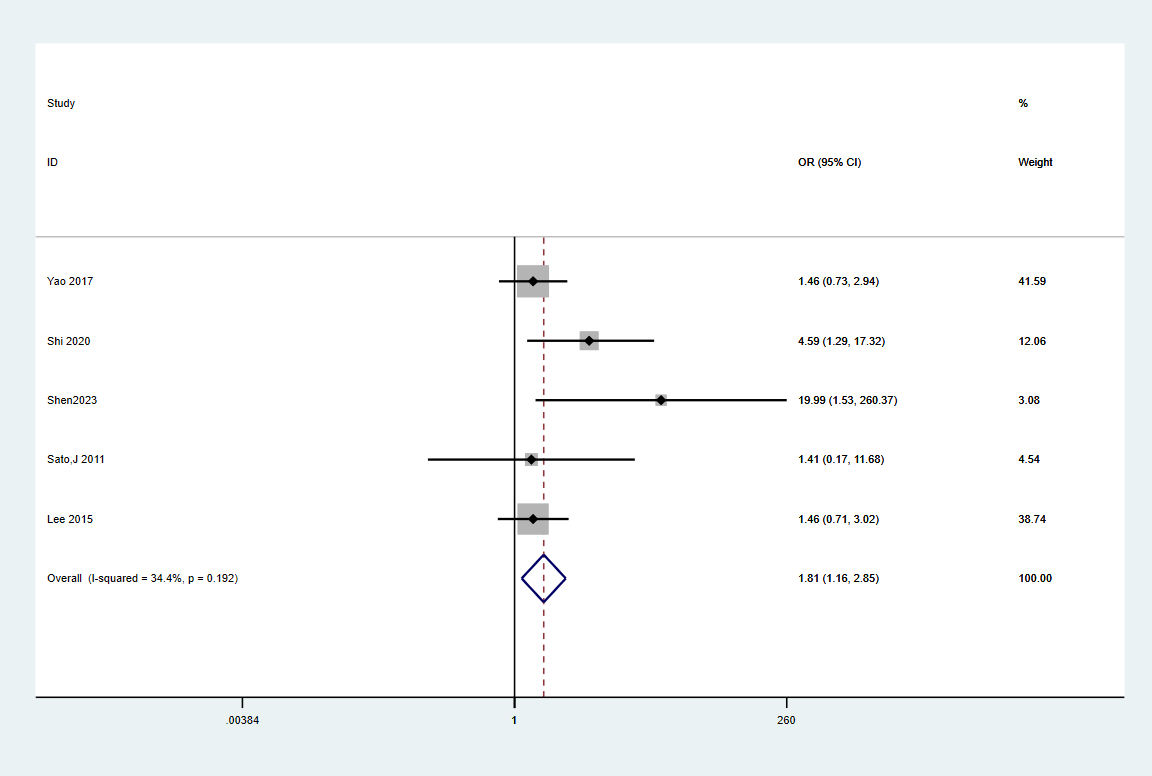


C Mandibulectomy


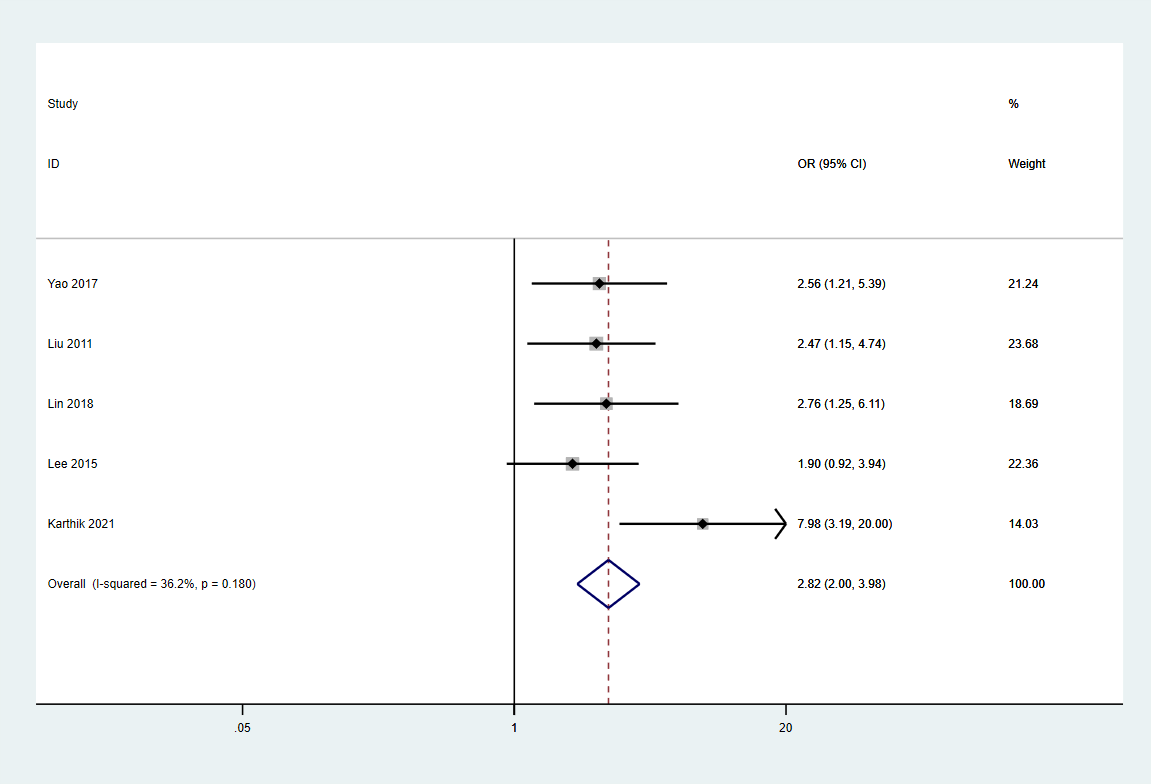


D diabetes


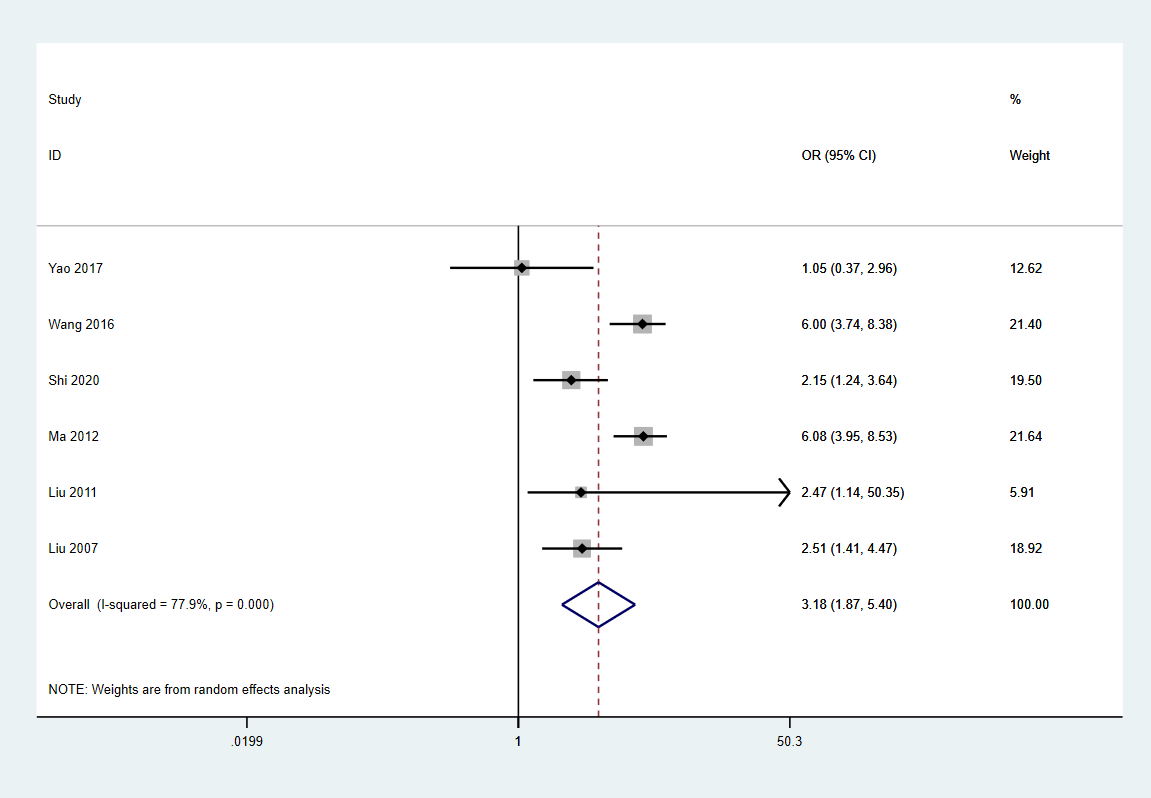


E BMI


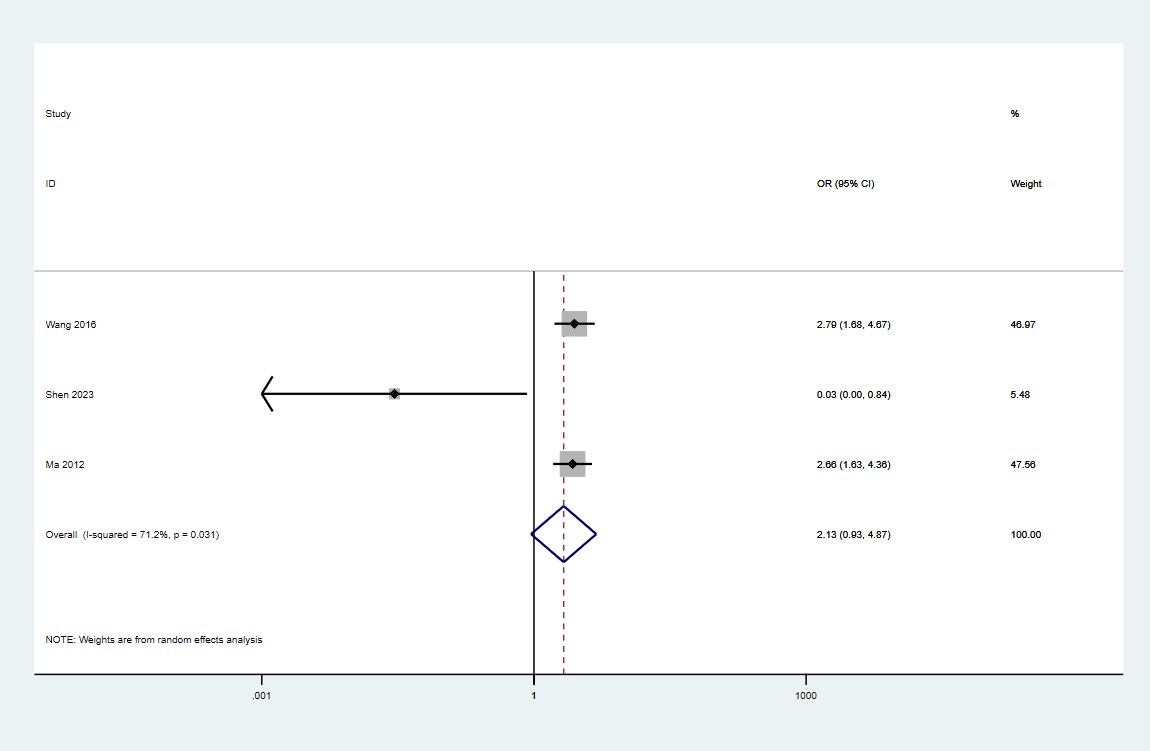


F ASA score


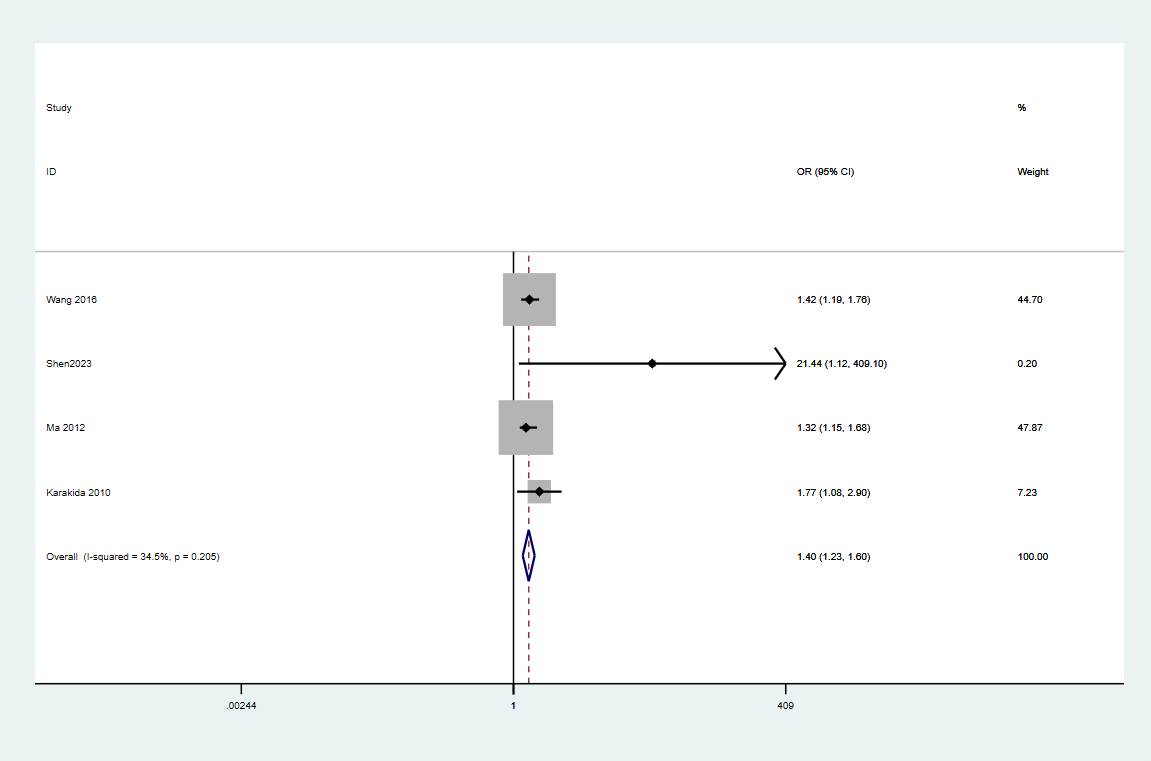


J ACE-27


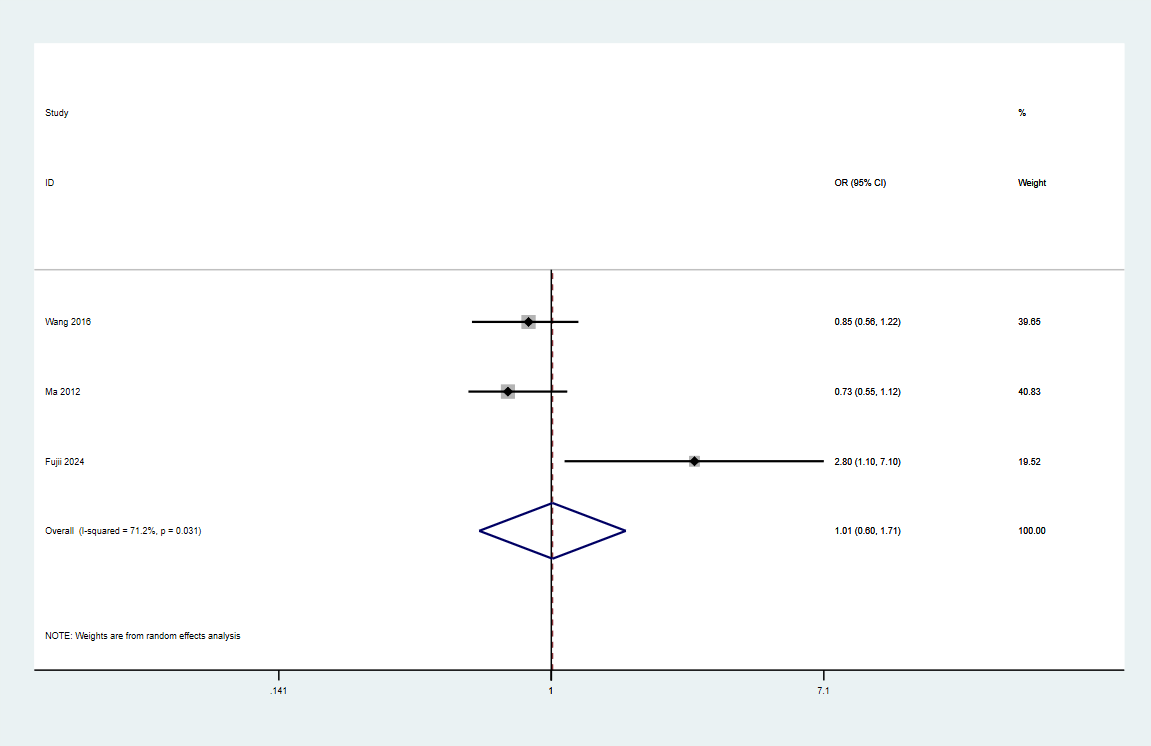


H operative time

I reconstructive method


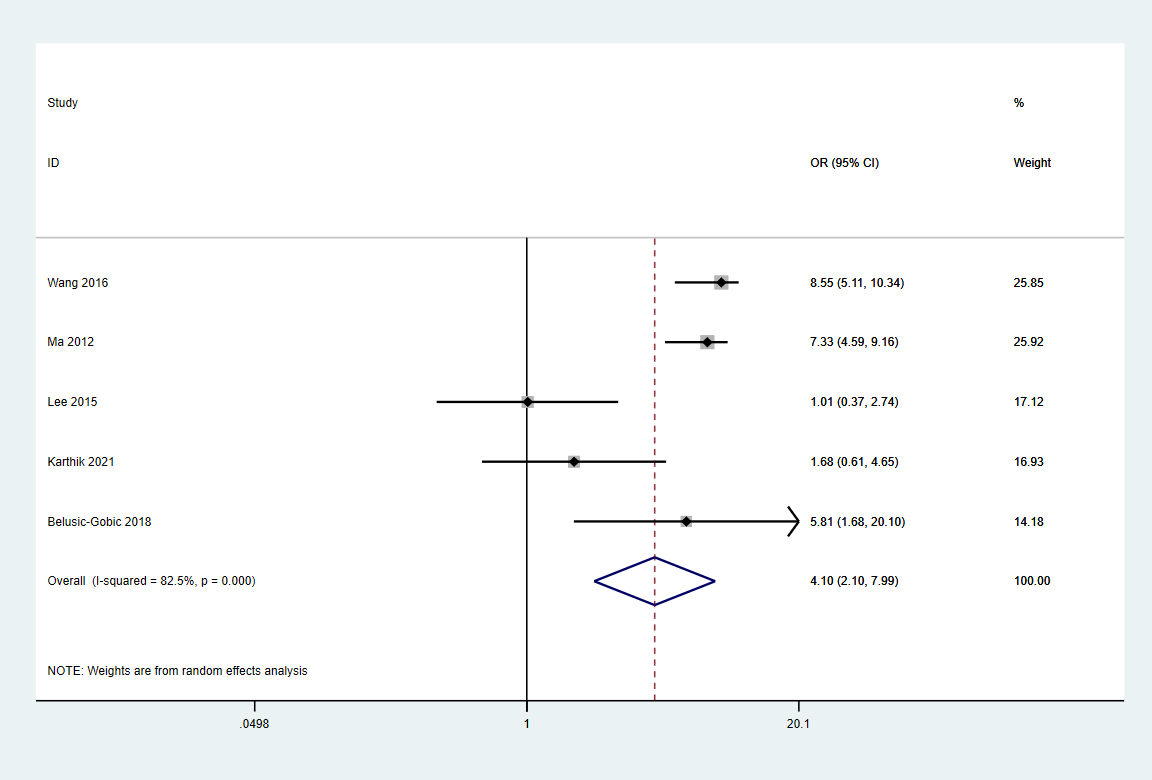


J Tracheostomy


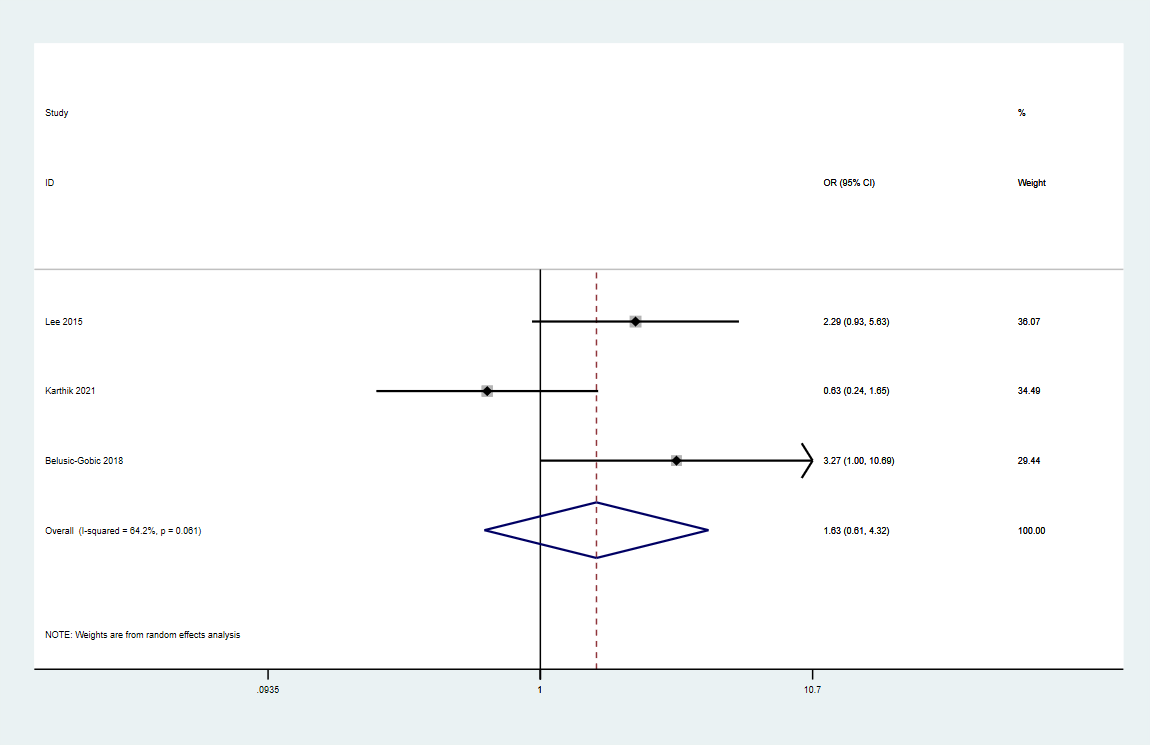


K transfusion


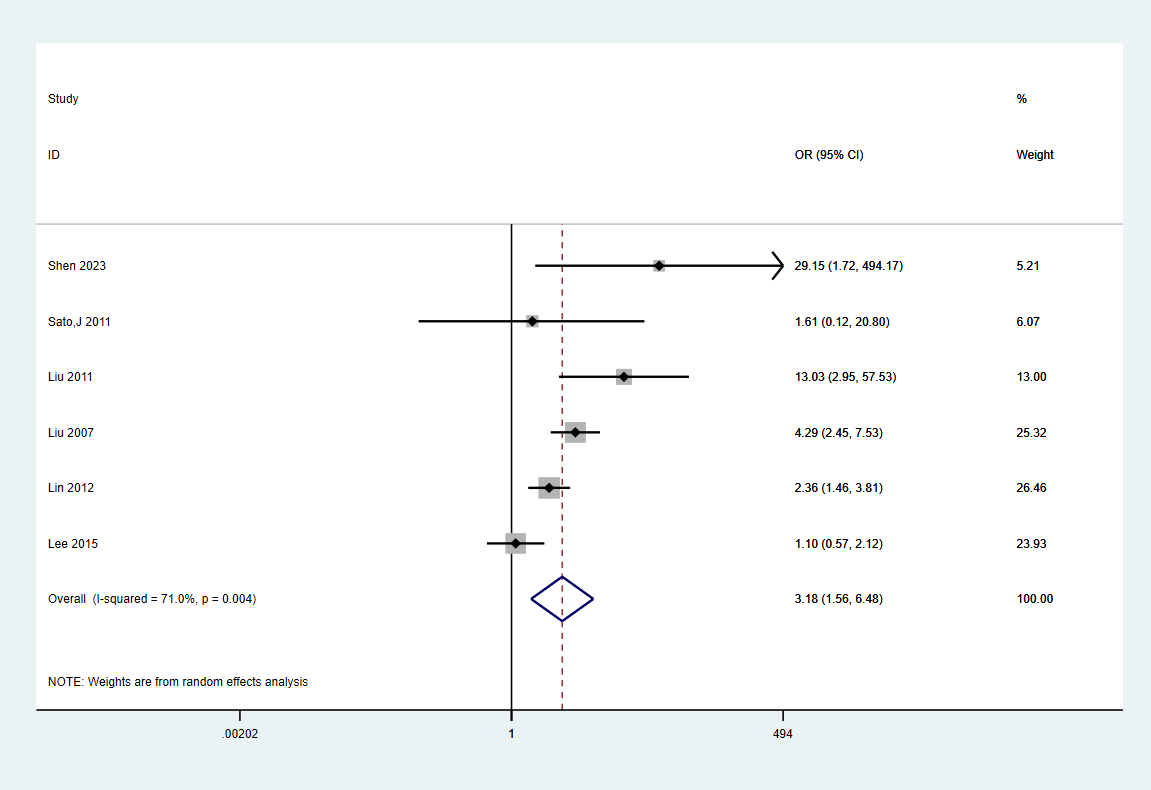


L Neck dissection


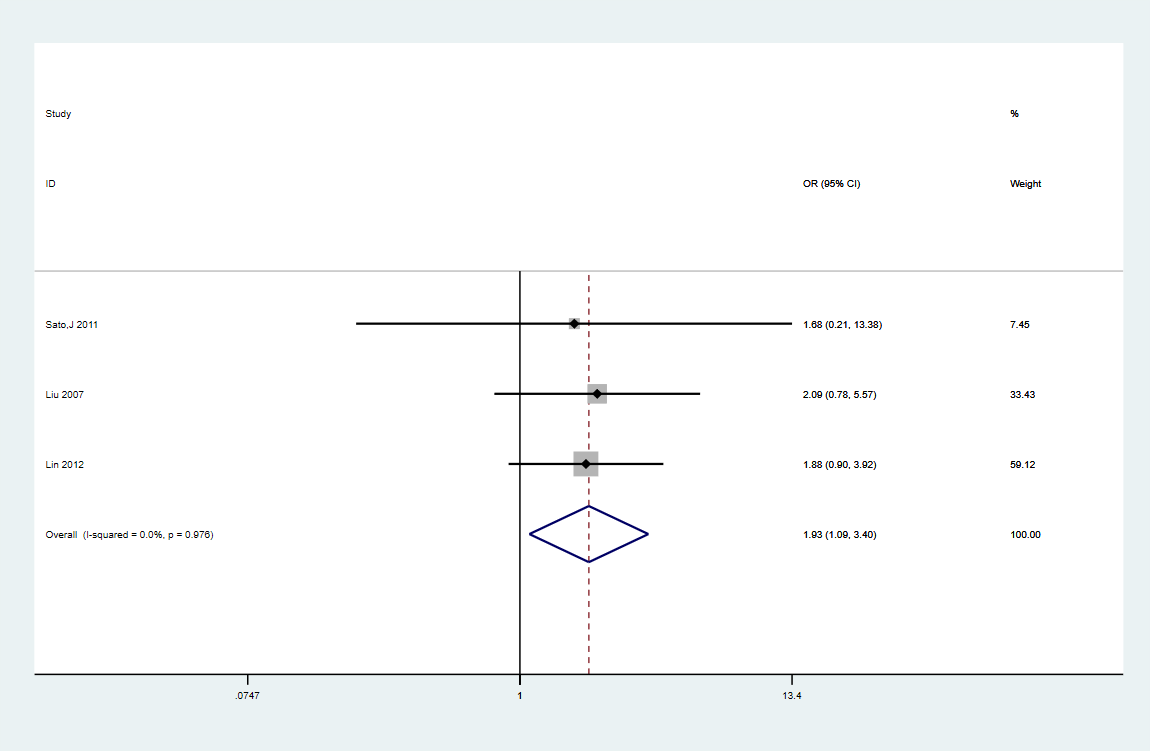


M Lower SMI


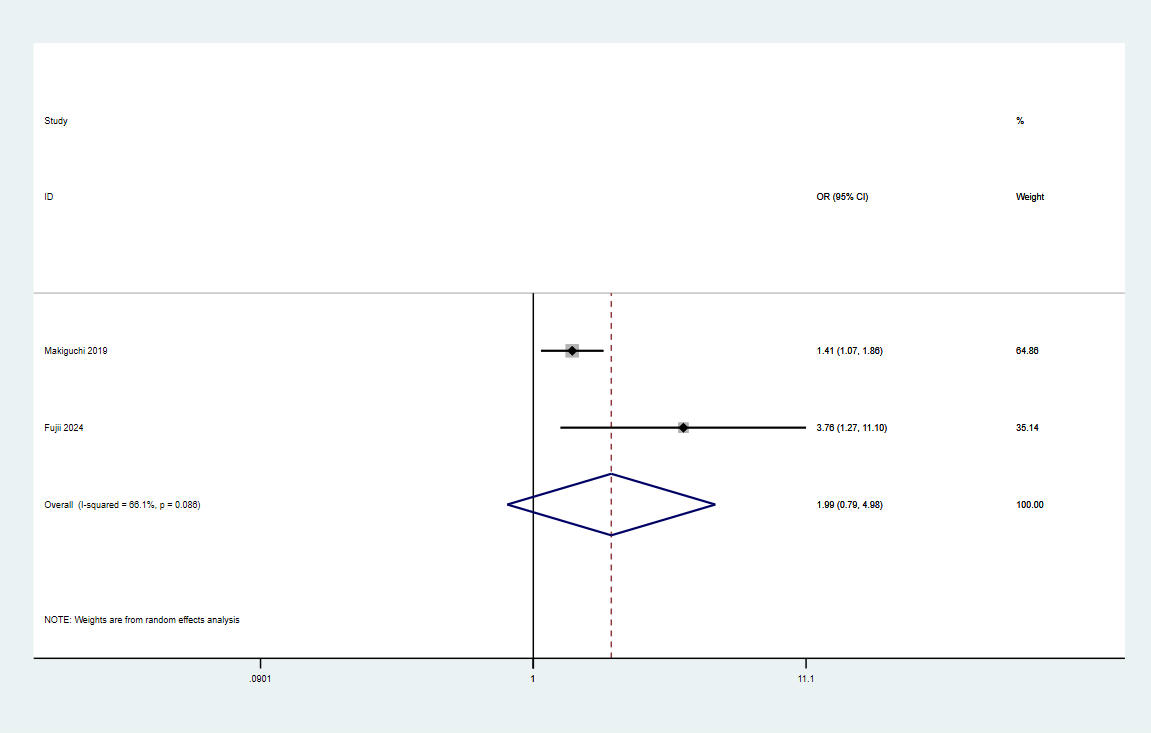


N albumin


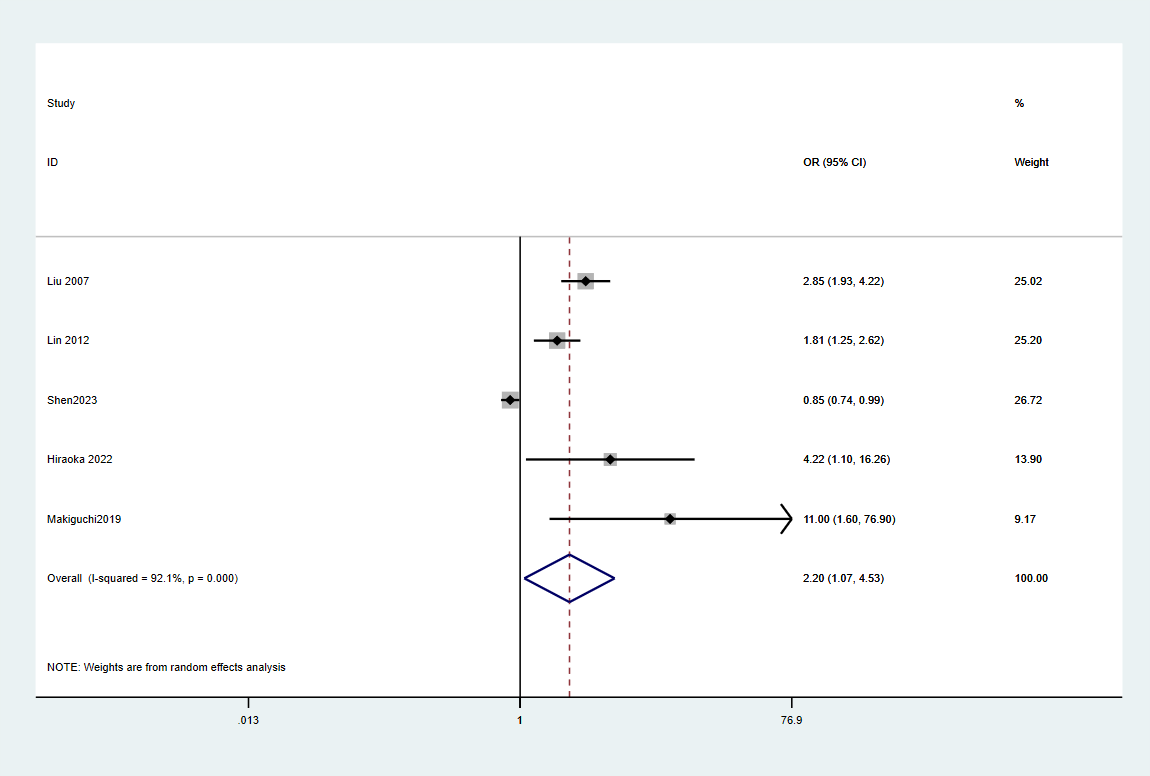


FIGURE S2 Forest plot of risk factors: A male, B radiotherapy, C Mandibulectomy, D diabetes, E BMI, F ASA score, G ACE-27, H operative time, I reconstructive surgery, J Tracheostomy,

K transfusion, L Neck dissection, M Lower SMI, N albumin

Table S1 Search strategy in Pubmed

| NO. | Query | Results |
| --- | --- | --- |
| #9 | #7 AND #8 | 428 |
| #8 | #5 OR #6 | 47,674 |
| #7 | #1 OR #4 | 199,838 |
| #6 | "Surgical Wound Infections"[Title/Abstract] OR "Surgical Site Infection"[Title/Abstract] OR "Postoperative Wound Infection"[Title/Abstract] | 14,292 |
| #5 | "Surgical Wound Infection"[Mesh] | 41,282 |
| #4 | #2 AND #3 | 157,197 |
| #3 | cancer*[Title/Abstract] OR carcinoma*[Title/Abstract] OR Neopla*[Title/Abstract] OR tumor*[Title/Abstract] OR malignan*[Title/Abstract] | 4,061,838 |
| #2 | Mouth*[Title/Abstract] OR Oral*[Title/Abstract] OR Oropharyn*[Title/Abstract] | 924,842 |
| #1 | "Mouth Neoplasms"[Mesh] | 78,368 |

Table S2 Search strategy in Embase

| NO. | Query | Results |
| --- | --- | --- |
| #9 | #5 AND #8 | 893 |
| #8 | #6 OR #7 | 73251 |
| #7 | 'surgical site infection':ab,ti OR 'surgical wound infection':ab,ti OR 'postoperative wound infection':ab,ti OR 'surgical infection':ab,ti | 20727 |
| #6 | 'surgical infection'/exp | 69439 |
| #5 | #1 OR #4 | 312908 |
| #4 | #2 AND #3 | 237129 |
| #3 | cancer*:ab,ti OR carcinoma*:ab,ti OR neopla*:ab,ti OR tumor*:ab,ti OR malignan*:ab,ti | 5506342 |
| #2 | mouth*:ab,ti OR oral*:ab,ti OR oropharyn*:ab,ti OR 'oral cavity':ab,ti OR 'buccal mucosa':ab,ti OR 'intraoral':ab,ti OR 'mouth cavity':ab,ti | 1293096 |
| #1 | 'mouth tumor'/exp | 138230 |

Table S3 Search strategy in Cochrane library

| NO. | Query |
| --- | --- |
| #9 | #7 AND #8 |
| #8 | #5 OR #6 |
| #7 | #1 OR #4 |
| #6 | (Surgical Site Infection):ti,ab,kw OR (Postoperative Wound Infection):ti,ab,kw OR (surgical wound infection):ti,ab,kw OR (surgical infection):ti,ab,kw |
| #5 | MeSH descriptor: [Surgical Wound Infection] explode all trees |
| #4 | #2 AND #3 |
| #3 | (cancer*):ti,ab,kw OR (carcinoma*):ti,ab,kw OR (Neopla*):ti,ab,kw OR (tumor*):ti,ab,kw OR (malignan*):ti,ab,kw |
| #2 | (Mouth*):ti,ab,kw OR (Oral*):ti,ab,kw OR (Oropharyn*):ti,ab,kw OR (‘oral cavity’):ti,ab,kw OR (‘buccal mucosa’):ti,ab,kw OR (‘intraoral’):ti,ab,kw OR ('mouth cavity’):ti,ab,kw |
| #1 | MeSH descriptor: [Mouth Neoplasms] explode all trees |

Table S4 Search strategy in Web of Science

| NO. | Query | Results |
| --- | --- | --- |
| #5 | #3 AND #4 | 292 |
| #4 | TS=(“surgical site infection” OR “surgical wound infection” OR “postoperative wound infection” OR “surgical infection”) | 17469 |
| #3 | #1 AND #2 | 145636 |
| #2 | TS=( cancer* OR carcinoma* OR Neopla*OR tumor* OR malignan*) | 3991472 |
| #1 | TS = (Mouth* OR Oral* OR Oropharyn*OR "oral cavity" OR "buccal mucosa" OR "intraoral" OR "mouth cavity") | 980404 |
